# Activation of microRNA-378a-3p biogenesis promotes hepatic secretion of VLDL and hyperlipidemia by modulating ApoB100-Sortilin1 axis

**DOI:** 10.7150/thno.39578

**Published:** 2020-03-04

**Authors:** Tianpeng Zhang, Hongtao Shi, Ningning Liu, Jing Tian, Xiaoling Zhao, Clifford J. Steer, Qinghua Han, Guisheng Song

**Affiliations:** 1 Department of Medicine, University of Minnesota Medical School, Minneapolis, Minnesota 55455; 2 Department of Cardiology, the First Hospital of Shanxi Medical University, Taiyuan City, China 030001; 3 MC Lab, South San Francisco, CA 94080

**Keywords:** microRNA, hyperlipidemia, lipoprotein

## Abstract

**Rationale**: Hyperlipidemia is a major risk factor of atherosclerosis and cardiovascular diseases (CVD). As a standard-of-care approach for hyperlipidemia, statins only reduce the risk of coronary artery disease by 20-40%, underscoring the importance of identifying molecular pathways for the design of drugs against this disorder. Alterations in microRNA (miRNA) expression have been reported in patients with hyperlipidemia and CVD. This study was designed to determine the mechanism of dysregulated miR-378a-3p under the status of hyperlipidemia and evaluate how miR-378a-3p regulates hepatic secretion of VLDL.

**Methods**: Wild-type mice kept on a high fat diet were injected with miR-378a-3p inhibitor or a mini-circle expression system containing miR-378a precursor to study loss and gain-of functions of miR-378a-3p. Mice were treated with Triton WR1339 and ^35^S-methionine/cysteine to determine the effect of miR-378a-3p on hepatic secretion of VLDL. Database mining, luciferase assay, and ChIP (chromatin immunoprecipitation) were used to study the mechanism of dysregulated miR-378a-3p biogenesis.

**Results**: miR-378a-3p expression is significantly increased in livers of hyperlipidemic mice. *Sort1* (sortilin 1) was identified as a direct target of miR-378a-3p. By inhibiting the function of sortilin 1 as a transmembrane trafficking receptor, miR-378a-3p stabilized ApoB100 and promoted ApoB100 secretion *in vitro*. Liver-specific expression of miR-378a-3p stabilized ApoB100 and facilitated hepatic secretion of VLDL, which subsequently increased levels of VLDL/LDL cholesterol as well as triglycerides. In contrast, antagonizing miR-378a-3p using its inhibitor increased hepatic expression of *Sort1* and reduced hepatic export of VLDL with its consequent effects of serum lipid levels. Additional knockdown of up-regulated *Sort1* in livers of mice offset the effects of miR-378a-3p inhibitor, suggesting that *Sort1* was indispensable for miR-378a-3p to promote secretion of VLDL and thereby high levels of circulating VLDL/LDL cholesterol and triglycerides. Furthermore, oncogenic E2F1 (E2F transcription factor 1) was identified as a transcriptional activator of miR-378a-3p. *E2f1* knockdown, through reducing miR-378a-3p, impaired secretion of VLDL and reduced levels of VLDL/LDL cholesterol and triglycerides.

**Conclusions**: This study defines a novel pathway of E2F1-miR-378a-3p-SORT1-ApoB100 that controls levels of circulating VLDL/LDL cholesterol and triglycerides by modulating degradation and secretion of ApoB100, and suggests the use of miR-378a-3p as a potential therapeutic target for dyslipidemia.

## Introduction

Cardiovascular disease (CVD) is the leading cause of death in developed countries 1. Dyslipidemia has been shown to be one of the most potent risk factors for CVD 1, 2 and is characterized by elevated plasma levels of cholesterol and triglycerides (TG), and particularly LDL cholesterol. For the past 25 years, the statin (3-hydroxy-3-methylglutaryl CoA reductase inhibitors) class of cholesterol-lowering drugs has been used for the treatment of hypercholesterolemia, either alone or in combination with other classes of lipid-lowering drugs 3, 4. By inhibiting cholesterol synthesis and lowering LDL cholesterol, statins have been proven to be effective in reducing cardiovascular risks 5, 6. Despite aggressive use of statins, many individuals do not achieve the LDL cholesterol levels recommended by clinical guidelines 7. Inhibitors of PCSK9 (proprotein convertase subtilisin/kexin type 9) are a class of recently approved monoclonal antibody drugs that exhibit the therapeutic effect of high LDL cholesterol 8. However, safety concerns, high cost, and negligible effects on cardiovascular mortality limited its application 9, 10. New and effective therapeutic approaches to treating hyperlipidemia and CVD are not only necessary but urgently required.

The pathogenesis of hyperlipidemia and CVD involves many genetic and epigenetic alterations. It is well-established that the liver plays a critical role in regulating levels of circulating cholesterol and TG. This occurs primarily by their secretion and the transport of these lipids via VLDL and LDL 11. The ability of the liver to synthesize and secrete VLDL is regulated by availability of lipids or apolipoproteins that comprise the surface of VLDL 11, 12. Increased secretion of hepatic VLDL is one of the major causes of dyslipidemia 13. However, the underlying mechanisms of hepatic overproduction of VLDL and its secretion are not fully understood.

The discovery of a class of naturally-occurring small non-coding RNAs, termed microRNAs (miRNAs), has stimulated a new field of research on hyperlipidemia 14. Alterations in miRNA expression have been reported in patients with hyperlipidemia and CVD 15. Reflective of their key roles in lipid, lipoprotein and glucose metabolism (16, 17), miRNAs have been suggested as novel therapeutic targets for metabolic disorders. However, how miRNAs regulate the secretion of VLDL is poorly described. We observed that expression of miR-378a-3p was significantly increased in fatty livers of hyperlipidemic mice 16. Furthermore, we identified *Sort1* as a direct target of miR-378a-3p. *Sort1* encodes sortilin 1 that functions as an intracellular sorting receptor for apoB100 13. GWAS (genome wide association study) identified a common noncoding polymorphism in the locus encoding *Sort1* that is strongly correlated with high risk of CVD 17. Ablation of *Sort1* leads to increased plasma LDL/VLDL cholesterol by modulating hepatic VLDL secretion 17, indicating that miR-378a-3p-SORT1 axis is an important axis to regulate lipoprotein metabolism.

As reported previously, miR-378a-3p is embedded within the first intron of *Ppargc1β* 18. *Ppargc1β* encodes peroxisome proliferator-activated receptor γ coactivator 1 (PGC1β), a transcriptional coactivator that interacts with a broad range of transcription factors 19. It is well-established that PGC1β is a master regulator of lipid metabolism 20. Both genomic location and sequence of miR-378a-3p are conserved between human and mouse. Although miR-378a-3p is located within the first intron of *Ppargc1β*, we established that miR-378a-3p has its own transcription machinery instead of sharing the promoter with its host gene *Ppargc1β* 21. Increased miR-378a-3p in fatty livers urged us to search the promoter of miR-378a-3p and identified E2F1 as a potential transcription activator of miR-378a-3p. E2F1 is an oncogenic transcription factor that controls cell cycle 22. Recently, it was reported that E2F1 can increase lipogenesis and promote the development of hepatosteatosis 23. Based on our preliminary findings and those of others, we hypothesized that a pathway consisting of E2F1, miR-378a-3p, SORT1 and ApoB100 regulates hepatic secretion of VLDL. Upon activation, this axis maintains activation of miR-378a-3p biogenesis, suppression of *Sort1*, increased stabilization and secretion of ApoB100, which subsequently facilitates hepatic secretion of VLDL and aggravates the pathogenesis of hyperlipidemia. Both mouse models and cell lines were used to test our hypothesis.

## Materials and Methods

### Statement on institutional approval for mice experimentation

Eight-week-old wild-type male C57BL/6J mice (Jackson Laboratory) were used for experiments. Mice were housed in a barrier facility on 12 h:12 h light cycle with free access to water and a standard diet (SD) (Open Source D12450B: 10% Kcal fat) or a HFD (Open Source D12492: 60% Kcal fat). Animal care, plasmid injection, and surgical procedures were conducted in compliance with an approved IACUC protocol by University of Minnesota.

### Establishment of hyperlipidemic mice

Eight-week-old wild-type male C57BL/6 mice (Jackson Laboratory, *n*=6) were maintained on the SD (Open Source D12450B) or the HFD (Open Source D12492) for 8 weeks. After 8 weeks of HFD administration, livers and blood were collected for further analysis. Mice were housed, fed, and monitored in accordance with protocols approved by the committee for animal research at the University of Minnesota.

### Preparation of mini-circle expression vectors for miR-378a-3p and shRNA of *E2f1* or *Sort1*

We generated an *in vivo* expression vector of miR-378a-3p by cloning mouse miR-378a-3p precursor into mini-circle vectors purchased from System Biosciences (Cat. MN511A-1). A transthyretin gene (TTR) promoter was inserted into the upstream of miR-378a-3p precursor to ensure liver-specific expression of miR-378a-3p (MC-*TTR*- miR-378a). To rule out non-specific effects of the plasmid, we generated a miR-378a-3p mismatched-expression vector by mutating the seed region of miR-378a-3p, termed MC-*TTR*- miR-378a-MM. We inserted *E2f1* or *Sort1* shRNA into a mini-circle vector and the *TTR* promoter was used to ensure hepatic expression of *E2f1* or *Sort1* shRNA. This vector was referred as to MC-*TTR*-E2f1shRNA or MC-*TTR*-Sort1shRNA. Parental MC-*TTR*-miR-378-3p or MC-*TTR*-E2f1shRNA vector was transformed into a special host *E. coli* bacterial strain ZYCY10P3S2T (System Biosciences, Cat: MN900A-1). Mini-circles were generated based on the manufacturer's instructions.

### MC-*TTR*-miR-378a treatment of hyperlipidemic mice

Mice received a dose of 1.5 μg/g MC-*TTR*-miR-378a or control vector (MC-*TTR*-miR-378a-MM) complexed with *in vivo*-jetPEI (Polyplus Transfection, Strasbourg, France) weekly for eight weeks via tail-vein injection. At that time point, mice were anesthetized, and blood was collected by way of cardiac puncture. Subsequently, the livers were harvested and immediately frozen in liquid nitrogen for gene expression and histological analysis.

### Identification of miR-378 targets

To identify genes with binding motifs for miR-378a-3p, we downloaded the target gene database of Ago HITS-CLIP (high-throughput sequencing of RNAs isolated by crosslinking immunoprecipitation (HITS-CLIP) from Argonaute protein complex) 24, 25. We next performed mining of this database and identified that miR-378a-3p can physically bind to the 3'UTR of both human and mouse *Sort1* (Table S1). PITA software further confirmed that binding sites of miR-378a-3p within the 3'UTR of both human and mouse *Sort1* (Table S2) 26.

### Triton WR1339 treatment of mice

After four-hour fast, mice were injected intraperitoneally with Triton WR1339 (500 mg/kg) and 500 µCi of ^35^S-methionine/cysteine in saline to inhibit lipolysis and to label newly synthesized proteins. Plasma VLDL clearance is completely inhibited under these conditions 27, allowing us to determine the hepatic production rate of VLDL. Therefore, blood was taken before injection (50 μL) and at 1 hour after Triton WR1339 injection and plasma TG levels were assayed using an enzyme method (Wako, Osta, Japan). The VLDL-TG production rate was calculated by the increase in plasma TG level from baseline to 1 hour after Triton WR1339 injection. We used the 1 hour time point to estimate the rate of VLDL-TG production, assuming a linear increase of plasma TG concentration during this period. The data were expressed as micromoles of TG produced per hour per kilogram of body weight, assuming a plasma volume of 3.5% (liters per kilogram). VLDL was isolated by ultracentrifugation. Isolated VLDL was run on an SDS-PAGE gel and apoB bands were cut and counted for quantitation of VLDL secretion.

### Intravenous injection of miR-378a-3p-ASO and miR-378a-3p-MM-ASO

Both miR-378a-3p-ASO and miR-378a-3p-MM-ASO oligonucleotides (Exiqon) contained a fully phosphorothioate-modified backbone. To prevent toxicity and facilitate efficient cellular uptake, short (< 16mer) miRNA ASOs were constructed. The sequence of miR-378a-3p-MM-ASO was identical to miR-378a-3p-ASO, except for 4 base-pair changes that prevented binding to miR-378a-3p (T*G*ACAC*AA*GCT*CCA*G* vs T*G*ACTC*CA*AGT*CCA*G*). High target affinity was ensured by LNA modifications, as identified by asterisks in the sequences of miR-378a-3p-ASO and miR-378a-3p-MM-ASO. Both oligonucleotides were purified by reverse-phase high performance liquid chromatography and lyophilized. Lyophilized miR-378a-3p-ASO was re-suspended in NaCl 0.9% to a final concentration of 30 µg/µL. A dose of 25 µg/g body weight miR-378-3p-ASO in a total volume of 100 µL NaCl 0.9% was injected via the tail vein into C57BL/6 mice. Specifically, Eight-week old wild-type C57BL/6 mice were maintained on HFD for 8 weeks. At 16 weeks of age, mice were divided into three groups: Group I (control, n=9) were injected with a combination of 25 μg/g miR-378a-3p-MM-ASO (MM-ASO) and MC-TTR-miR-378a-3p-MM (1.5 μg/g); Group II (n=9) was injected with 25 μg/g miR-378a-3p-ASO and MC-TTR-miR-378a-3p-MM. This group was used to determine loss-of function of miR-378a-3p; and Group III (n=9) received a combination of 25 μg/g miR-378a-3p-ASO and MC-TTR-Sort1-shRNA (1.5 μg/g). This group was designed to knock down increased Sort1 due to miR-378a-3p-ASO treatment, allowing us to determine whether Sort1 mediates the inhibitory effect of miR-378a-3p-ASO on VLDL secretion.

### FPLC analysis

After five-hour fast, blood was collected into citrate-EDTA tubes. After plasma isolation by centrifugation, the distribution of lipids within the plasma lipoprotein fractions was assessed by fast phase liquid chromatography (FPLC). Pooled plasma from mice was used for FPLC analysis. Cholesterol levels of FPLC fractions were measured using Wako enzymatic kits (Cholesterol E).

### Metabolic labeling experiments

Dimethyl sulfoxide (DMSO), the proteasomal inhibitor MG132, and CP-10447 were obtained from Sigma (St. Louis, MO). The ^35^S-methionine/cysteine protein labeling mixture was obtained from Perkin- Elmer Life Sciences (Waltham, MA).

HepG2 cells were maintained in the high glucose DMEM containing 10% FBS and 0.5 mM oleate. Subconfluent HepG2 cells (80% confluency) were then rinsed twice with warm PBS, and preincubated for 60 min in experimental medium (DMEM without methionine/cysteine (Invitrogen), supplemented with 1% FBS, 0.5 mM oleate, 1% L-glutamine and penicillin/streptomycin). Cells were metabolically labeled with 120 μCi/mL of [^35^S]-methionine/cysteine for 3 hours at 37ºC in the presence of 25 μM MG132 or CP-10447. Media were collected and cell extract was obtained after incubation of cells in lysis buffer (50 mM Tris, pH 8.0, 150 mM NaC1, 0.02% sodium azide, 1 pg/mL aprotinin, and 1% NP-40) for 20 min. Cell extract and media were spun to remove cellular debris, and apoB was immune-precipitated. Immunoprecipitated samples were either directly counted in Ready SafeTM scintillation cocktail or run on a 3-13% SDS-PAGE gradient gel, dried, and exposed using autoradiography.

To study loss of function for miR-378a-3p in modulating ApoB100 stability and secretion, HepG2- miR cells were transfected with scramble control, miR-378a-3p-ASO or a combination of miR-378a-3p- ASO and MC-*TTR*-Sort1shRNA. 48 hours post transfection, cells were preincubated for 60 min in experimental medium (DMEM without methionine/ cysteine, supplemented with 1% FBS, 0.5 mM oleate, 1% L-glutamine and penicillin/streptomycin). Cells were metabolically labeled with 120 μCi/mL of [^35^S]-methionine/cysteine for 3 h at 37ºC in the presence of 25 μM MG132 or CP-10447. Media were collected and cell extract was obtained after incubation of cells in lysis buffer (50 mM Tris, pH 8.0, 150 mM NaC1, 0.02% sodium azide, 1 pg/mL aprotinin, and 1% NP-40) for 20 min. Cell extract and media were spun to remove cellular debris, and apoB was immunoprecipitated. Immunoprecipitated samples were either directly counted in Ready SafeTM scintillation cocktail or run on a 3-13% SDS-PAGE gradient gel, dried, and exposed using autoradiography.

### Statistical analysis

Statistical analysis was performed using GraphPad Prism Software®. Two-tailed Student *t* test, Mann-Whitney test, or ANOVA (analysis of variance) was used to evaluate the statistical significance. All *in vitro* experiments were repeated at least three times on separate occasions. Data were presented as mean ± SEM and *p* < 0.05 was considered statistically significant.

## Results

### *Sort1* is a direct target of miR-378a-3p whose expression is increased in fatty livers of hyperlipidemic mice

**Figure 1:**
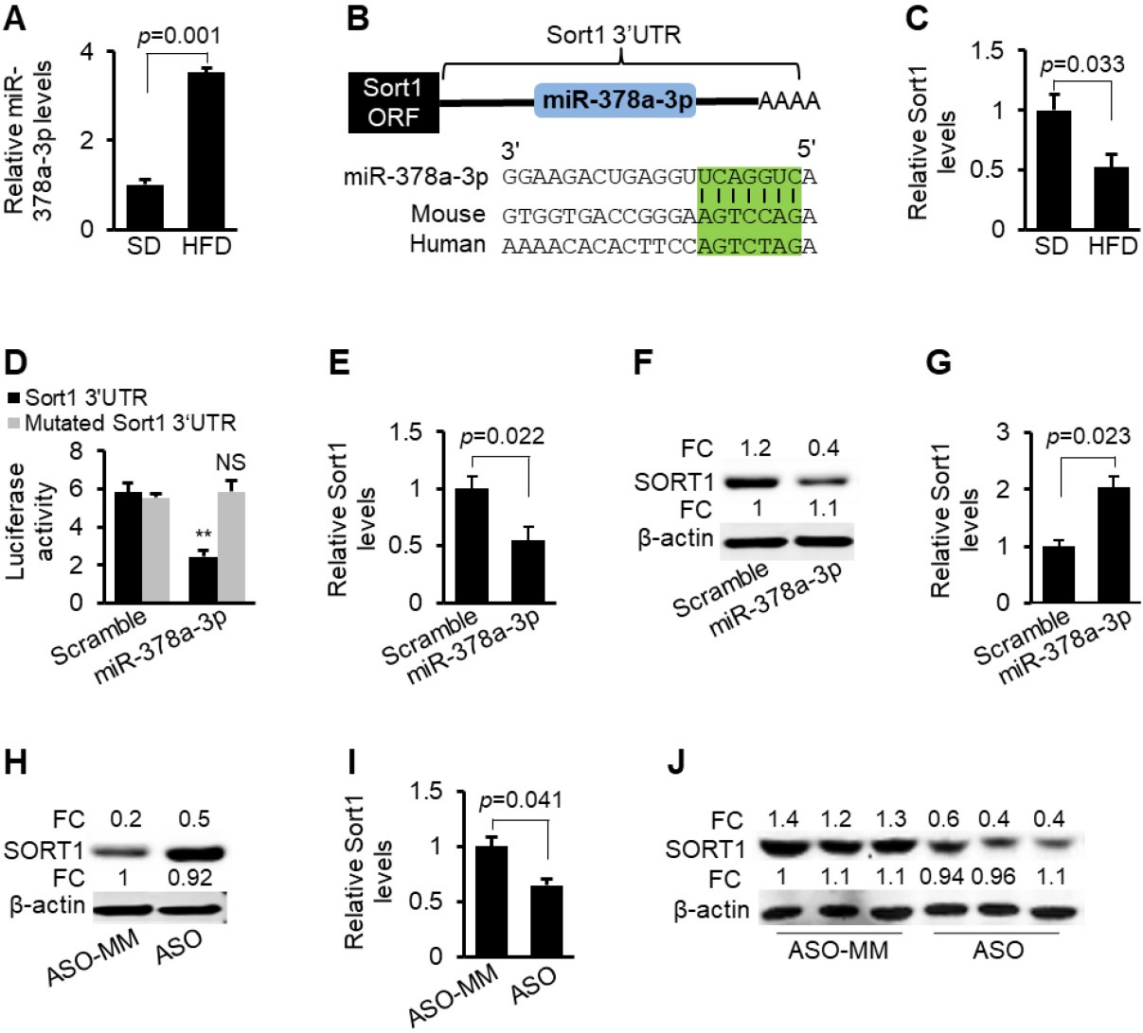
***Sort1* is a direct target of miR-378a-3p.** (**A**) Increased expression of miR-378a-3p in livers of HFD-fed mice (*n*=6) compared to SD-fed mice (*n*=6).** (B)** Graphic representation of the conserved miR-378a-3p binding motifs within the 3'UTR of *Sort1*. Complementary sequences to the seed regions of miR-378a-3p within the 3'UTRs are conserved between human and mouse (highlighted in green). G:U wobble is allowed during the prediction of PITA software. (**C**) Reduced mRNA levels of *Sort1* in livers of mice treated with HFD-(*n*=6) compared to SD-treated mice (control, *n*=6). (**D**) Luciferase activity of the luciferase reporter constructs containing either wild-type or mutated 3'UTR of *Sort1* after transfection of miR-378a-3p mimics or scramble (control treatment) into Hepa1-6 cells. NS: no significance. (**E-F**) mRNA and protein levels of *Sort1* in Hepa1-6 cells transfected with miR-378a-3p mimics or scramble (control). (**G-H**) Increased mRNA and protein levels of *Sort1* in Hepa1-6 cells transfected with miR-378a-3p-ASO versus miR-378a-3p-ASO-MM (ASO-MM, control). (**I-J**) Reduced protein and mRNA levels of *Sort1* after MC-*TTR*-miR-378a injection into dietary obese mice (*n*=6) versus MC-*TTR*-miR-378a-MM (n=6, control). Data represents mean ± SEM. **p < 0.01 (Student's* t* test).

In our previous study, we showed that miR-378a-3p was highly expressed in hepatocytes 28. Hepatocytes are the major cells that control lipid and lipoprotein metabolism. We, therefore, treated C57BL/6 mice with a HFD to induce hyperlipidemia and determined hepatic expression of miR-378a-3p. HFD treatment dramatically increased levels of hepatic and circulating cholesterol and TG (Figure S1A-C). miR-378a-3p expression was robustly increased in fatty livers of hyperlipidemia mice (Figure 1A). In dyslipidemia patients, levels of miR-378a-3p are also significantly increased 16, 29. All these findings suggest that miR-378a-3p was potentially involved in regulation of lipoprotein metabolism.

To gain insight into the function of miR-378a-3p, we attempted to identify target genes of miR-378a-3p by combining bioinformatic prediction and database mining of Ago HITS-CLIP 24, 25. HITS-CLIP database mining showed that miR-378a-3p had two binding sites within the 3'UTRs of *Sort1* (Table S1). Indeed, PITA software identified two miR-378a-3p binding sites within the 3'UTR of *Sort1* 26 (Figure 1B, Table S2).* Sort1* encodes the sortilin protein that functions as a multi-ligand sorting receptor 30. Sortilin localizes to various intracellular compartments including the Golgi apparatus and has roles in both endocytosis and intracellular trafficking of other proteins 31. Liver-specific expression of* Sort1* alters levels of plasma LDL/VLDL cholesterol by modulating hepatic secretion of VLDL 17. All these findings suggested that miR-378a-3p can promote hepatic VLDL secretion by inhibiting *Sort1*. qRT-PCR confirmed that *Sort1* had a decreased expression in livers of hyperlipidemic mice (Figure 1C), consistent with elevated miR-378 in fatty livers.

To establish that miR-378a-3p directly recognizes the predicted binding sites within the 3' UTR of *Sort1*, its 3' UTR was cloned into a luciferase reporter vector. Inclusion of the *Sort1* 3'UTR into a luciferase reporter construct reduced luciferase activity upon co-transfection with miR-378a-3p mimics into Hepa1-6 cells (Figure 1D). In contrast, mutation of the miR-378a-3p binding sites was necessary to completely offset the inhibitory effect of miR-378a-3p on luciferase activity (Figure 1D). miR-378a-3p also significantly inhibited expression of endogenous *Sort1* in Hepa1-6 cells (Figure 1E-F), while miR-378a-3p knockdown led to an opposite effect (Figure 1G-H). Delivery of miR-378a-3p into livers also repressed mRNA and protein levels of *Sort1* in mice (Figure 1I-J). Together, our data indicate that *Sort1* is a direct target of miR-378a-3p both *in vitro* and* in vivo*.

### Liver-specific expression of miR-378a-3p increased levels of circulating VLDL/LDL cholesterol and TG

**Figure 2:**
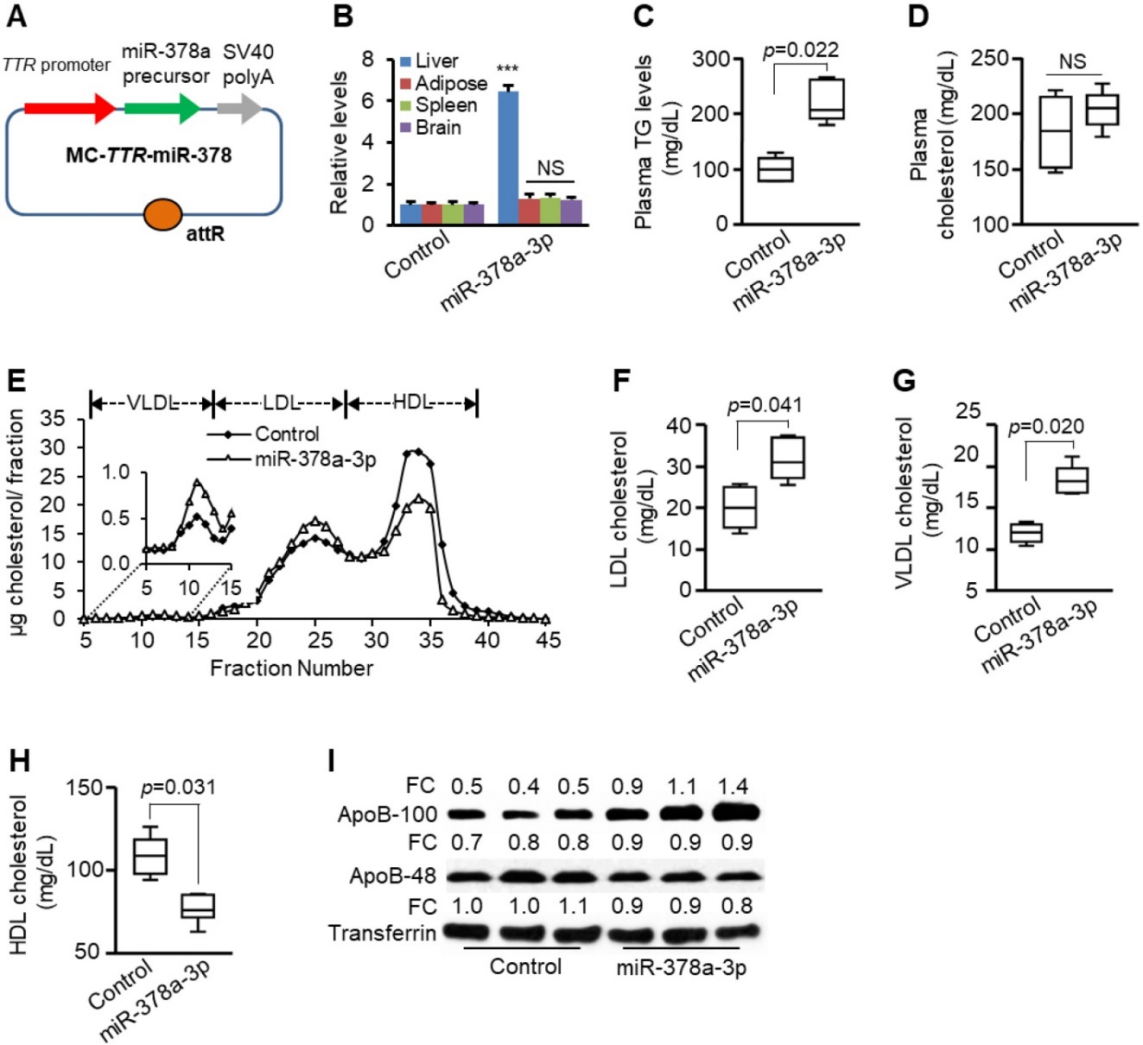
** Liver-specific expression of miR-378a-3p increased levels of plasma triglycerides and LDL/VLDL cholesterol.** (**A**) Diagram of hepatic-specific miR-378a expression construct. (**B**) Levels of miR-378a-3p in different organs four weeks after one injection of MC-*TTR*-miR-378a into mice (*n*=6) or MC-*TTR*-miR-378a-MM (control, *n*=6). (**C-D**) Levels of total plasma triglycerides and cholesterol in mice treated with MC-*TTR*-miR-378a or MC-*TTR*-miR-378a-MM (control). (**E**) FPLC profile of pooled plasma from two groups of mice treated with MC-*TTR*-miR-378a (*n*=9) or MC-*TTR*-miR-378a-MM (control, *n*=9). (**F-H**) MC-*TTR*-miR-378a treatment led to increased LDL/VLDL cholesterol and reduced HDL cholesterol (pooled FPLC). (**I**) Levels of ApoB100 and ApoB48 in the plasmas of the above two groups of mice. Data represent mean ± SEM. ***p < 0.001 (Mann-Whitney test).

Increased hepatic secretion of VLDL is a major cause of elevated LDL/VLDL cholesterol and TG 32. We assumed that miR-378a-3p could increase levels of circulating VLDL/LDL cholesterol and TG by facilitating hepatic VLDL secretion. Secreted VLDL is hydrolyzed by lipoprotein lipase (LPL) to VLDL remnant that can be further metabolized to LDL 33. Therefore, we next examined gain-of function for miR-378a-3p. For this purpose, mini-circle vector encoding the murine miR-378a-3p driven by a liver-specific promoter *TTR* (MC-*TTR*-miR-378a) was delivered into mice via tail-vein injection 34 (Figure 2A). Specifically, two groups of C57BL/6 wild-type mice on the HFD for eight weeks were injected with either MC-*TTR-*miR-378a-MM (control) or MC-*TTR-*miR-378a for eight weeks. Another group of mice maintained on standard diet (SD) that received MC-*TTR*-miR-378a-MM served as another control. Compared to SD-fed mice, HFD treatment led to increased hepatosteatosis and circulating TG and cholesterol (Figure S2A-B). Injection of MC-*TTR*-miR-378a resulted in increased miR-378a-3p in the liver without significant change in other organs (Figure 2B). MC-*TTR*-miR-378a treatment increased hepatosteatosis (Figure S2A-B), consistent with our previous publication showing that miR-378a-3p promotes hepatosteatosis by impairing fatty acid oxidation (FAO) 21. Compared with control mice, a marked increase in plasma TG was observed in mice treated with miR-378a-3p (Figure 2C), while plasma cholesterol showed no significant change (Figure 2D).

Compared with SD-fed mice, HFD treatment significantly increased levels of VLDL, LDL and HDL (Figure S2C). Considering the role of *Sort1* in modulating hepatic VLDL secretion 17, the lipoprotein profiles were compared between two groups of mice treated with miR-378a-3p-tretaed and control mice. As revealed by plasma fractionation analysis, liver-specific expression of miR-378a-3p significantly increased levels of LDL/VLDL cholesterol (Figure 2E-G). Unexpectedly, miR-378a-3p also reduced HDL cholesterol (Figure 2H), urging us to predict more targets of miR-378a-3p. As expected, we identified *ABCG1* gene encoding ATP-binding cassette transporters G1 as a direct target of miR-378a-3p (Figure S3). It is known that ABCG1 ablation reduced HDL cholesterol 35, indicating the potential contribution of miR-378a-3p-ABCG1 axis to reduced HDL cholesterol, which is beyond this study. ApoB is the major protein component of VLDL. Indeed, liver-specific expression of miR-378a-3p also increased plasma levels of ApoB100 (Figure 2I). Microsomal triglyceride transfer protein (MTP) is responsible for the hepatic assembly of VLDL particles through the transfer of TG to ApoB 36. However, both MTP activity and mRNA levels of *Mttp* and *ApoB100* were not altered after miR-378a-3p overexpression (Figure S4A-C). Activated hepatic lipogenesis is another factor that contributes to increased VLDL secretion and plasma TG 32. qRT-PCR revealed that miR-378a-3p had no effect on mRNA levels of *Srebp1c* and lipogenic genes including *Scd1* (stearoyl-CoA desaturase 1) and *Fasn* (fatty acid synthase) (Figure S4D). These findings indicate that MTP-mediated assembly of VLDL particles and hepatic lipogenesis are not involved in increased plasma VLDL/LDL cholesterol and TG by miR-378a-3p. Together, our results indicated that miR-378a-3p promotes hepatic secretion of VLDL, thereby increasing levels of plasma TG and LDL/VLDL cholesterol.

### Antagonizing miR-378a-3p reduced circulating LDL/VLDL cholesterol and triglycerides

**Figure 3:**
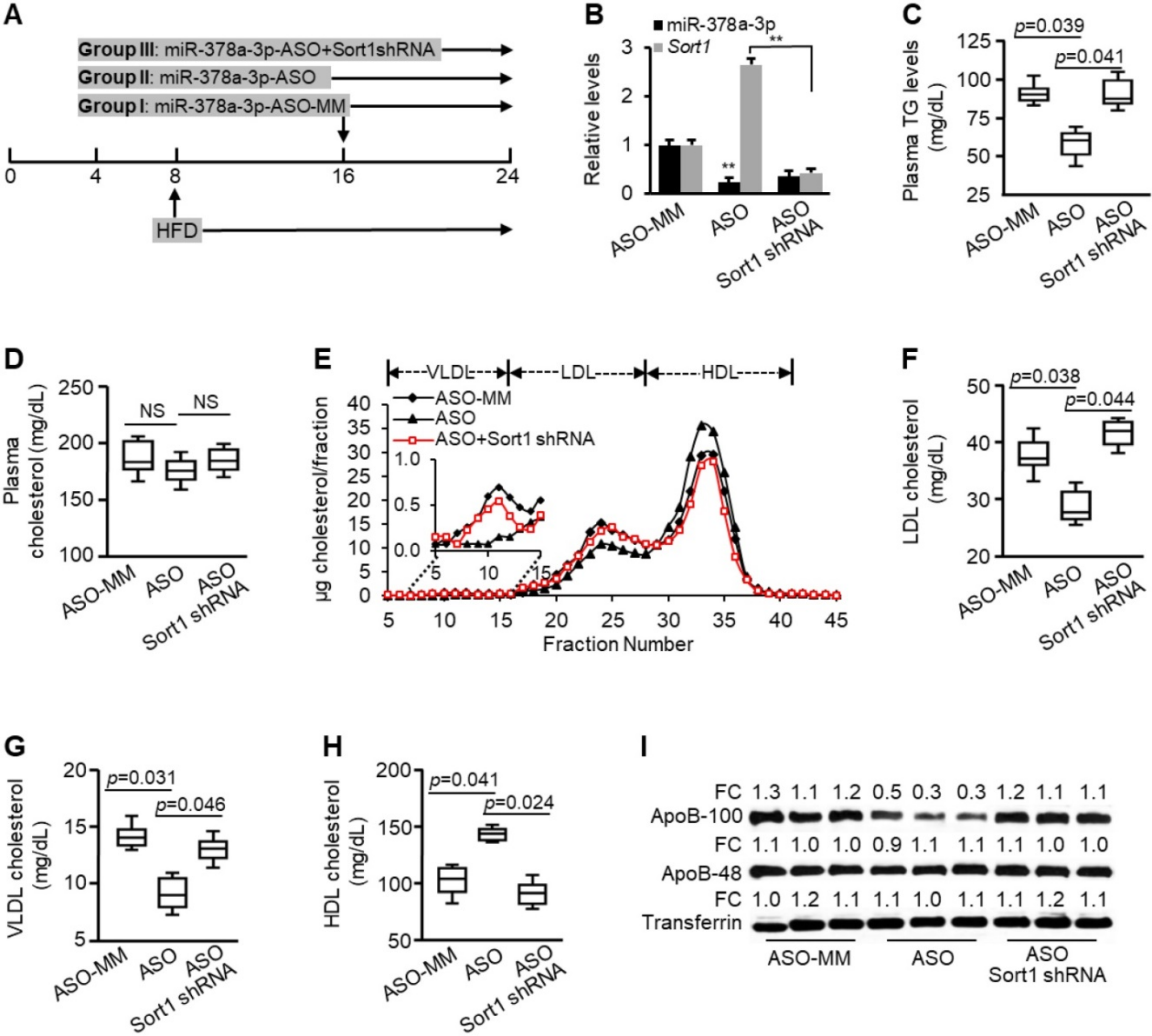
***Sort1* mediated the inhibitory effects of miR-378a-3p-ASO on plasma levels of triglyceride and LDL/VLDL cholesterol.** (**A**) Schematic representation of treatment of scramble (control, *n*=9), miR-378a-3p-ASO (*n*=9) and a combination of miR-378a-3p-ASO and MC-*TTR*-Sort1shRNA (*n*=9). (**B**) Levels of miR-378a-3p and *Sort1* mRNAs in livers of three groups of mice. (**C**) Levels of total triglyceride in pooled plasma from three groups of mice. (**D**) No change was observed in levels of total cholesterol of pooled plasma among three groups of mice. (**E**) FPLC profile of pooled plasma from three groups of mice. (**F-H**) Levels of LDL, VLDL and HDL cholesterol in pooled plasma from three groups of mice. (**I**) Levels of ApoB100 and ApoB48 in the pooled plasma of three groups of mice. Data represent mean ± SEM. **p < 0.01 ***p < 0.001 (ANOVA test).

We further determined loss-of function for miR-378a-3p. miRNAs can target many genes simultaneously 14. Therefore, we also determined whether *Sort1* mediates the role of miR-378a-3p in facilitating hepatic secretion of VLDL. After 8 weeks on the HFD, WT C57BL/6 mice were randomly allocated into three groups and received the different treatments for another eight weeks. Group I received scramble control (ASO-MM); Group II received miR-378a-3p-ASO to induce expression of *Sort1*; and Group III received a combination of miR-378a-3p-ASO and *Sort1* shRNA. Such a design allowed us to determine whether *Sort1* is required for miR-378a-3p-ASO to reduce plasma TG and LDL/VLDL cholesterol (Figure 3A). We observed a 70% reduction in hepatic miR-378a-3p expression in mice that had received miR-378a-3p-ASO compared to those receiving the scramble control, and a significant increase in *Sort1* mRNA (Figure 3B). A 31% reduction in plasma TG was observed in miR-378a-3p-ASO-treated mice (Figure 3C). Although no significant change in total cholesterol was observed (Figure 3D), miR-378 inactivation resulted in a significant decrease in LDL/VLDL cholesterol and an increase in HDL cholesterol (Figure 3E-H).

To investigate if *Sort1* mediates the inhibitory effects of miR-378a-3p-ASO on plasma TG and LDL/ VLDL cholesterol, we compared miR-378a-3p-ASO-treated mice with those treated with a combination of miR-378a-3p-ASO and *Sort1* shRNA. Additional treatment of *Sort1* shRNA knocked down increased *Sort1* by miR-378a-3p-ASO (Figure 3B). Phenotypically, additional knockdown of *Sort1* restored levels of plasma TG and LDL/VLDL cholesterol (Figure 3C, E-H), while no recovery in total plasma cholesterol was observed (Figure 3D). Consistent with reduced LDL/VLDL cholesterol, miR-378a-3p knockdown reduced plasma ApoB100, and additional treatment of *Sort1* shRNA counteracted the effect of miR-378a-3p-ASO (Figure 3I). Together, these findings indicated that *Sort1*, at least in part, mediated the inhibitory effects of miR-378a-3p-ASO on plasma TG and LDL/VLDL cholesterol.

### miR-378a-3p increased stability and secretion of ApoB100

ApoB100 is essential for the assembly of VLDL 32. SORT1 is involved in ApoB trafficking and degradation 37. We, therefore, hypothesized that miR-378a-3p increases ApoB100 stability and/or facilitate secretion of ApoB100 by targeting *SORT1*. Human hepatoma HepG2 cells have been used to study ApoB100 secretion 38, 39. To determine the effect of miR-378a-3p on the ApoB100 degradation and secretion, we established a HepG2 cell line that stably expresses miR-378a-3p (HepG2-miR).

**Figure 4:**
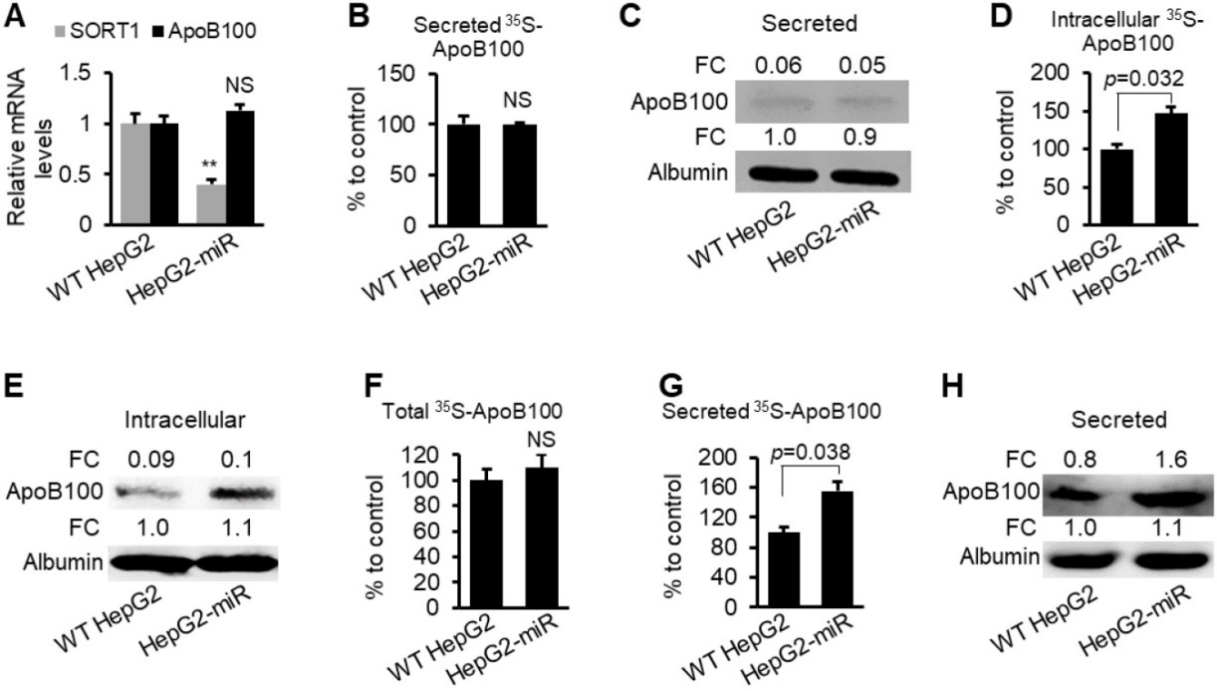
** miR-378a-3p prevented degradation of ApoB100 and facilitated secretion of ApoB100 in HepG2 cells.** (**A**) mRNA levels of *ApoB100* and *SORT1* in WT HepG2 (control) and HepG2-miR cells. (**B-C**) CP-10447 strongly inhibited ApoB100 secretion in both WT HepG2 and HepG2-miR cells, which was reflected by almost undetectable ^35^S-ApoB100 in the medium from WT HepG2 and HepG2-miR cells. WT HepG2 and HepG2-miR cells were starved for one hour in cysteine and methionine free medium and then labeled with ^35^S-methionine/cysteine for 3 hours in the presence of MTP inhibitor CP-10447. (**D-E**) Increased intracellular ^35^S-ApoB100 in HepG2-miR cells treated with CP-10447. (**F**) No significant change in levels of total ^35^S-ApoB100 (intracellular and medium) in WT HepG2 and HepG2-miR cells. WT HepG2 and HepG2-miR cells were starved for one hour in cysteine and methionine free median and then labeled with ^35^S-methionine/cysteine for 3 hours in the presence of MG132 (proteasome inhibitor). (**G**-**H**) Increased levels of secreted ApoB100 in the medium of HepG2-miR cells versus WT HepG2 cells treated with MG132. Data represent mean ± SEM. **p < 0.01 (Student *t* test).

CP-10447, an inhibitor of MTP, can inhibit ApoB secretion from HepG2 cells 38, 39, allowing us to study the role of miR-378a-3p in modulating degradation of ApoB. To test the role of miR-378a-3p in ApoB100 degradation, HepG2-miR and wild-type (WT) HepG2 cells were labeled with ^35^S-methionine in the presence of CP-10447. miR-378a-3p reduced expression of *SORT1* but did not change mRNA levels of *ApoB100* (Figure 4A). CP-10447 blocked ApoB100 secretion in both WT HepG2 and HepG2-miR cells, which was reflected by nearly undetectable ApoB100 in the medium (Figure 4B-C). However, levels of intracellular radioactive ApoB100 are significantly higher in HepG2-miR compared with WT HepG2 cells (Figure 4D-E), indicating that miR-378a-3p increased stability of ApoB100. To determine whether miR-378a-3p facilitates secretion of ApoB100, we treated WT HepG2 and HepG2-miR cells with the proteasome inhibitor MG132 that inhibits degradation of ApoB100. Such a design excluded the effect of ApoB100 degradation and allowed us to determine the effect of miR-378a-3p on ApoB100 secretion. No significant change in levels of total radioactive ApoB100 was observed between WT HepG2 and HepG2-miR cells (Figure 4F), while levels of radioactive ApoB100 was significantly increased in the medium of HepG2-miR cells (Figure 4G-H). All these findings indicated that miR-378a-3p is able to stabilize ApoB100 and facilitate its secretion.

**Figure 5:**
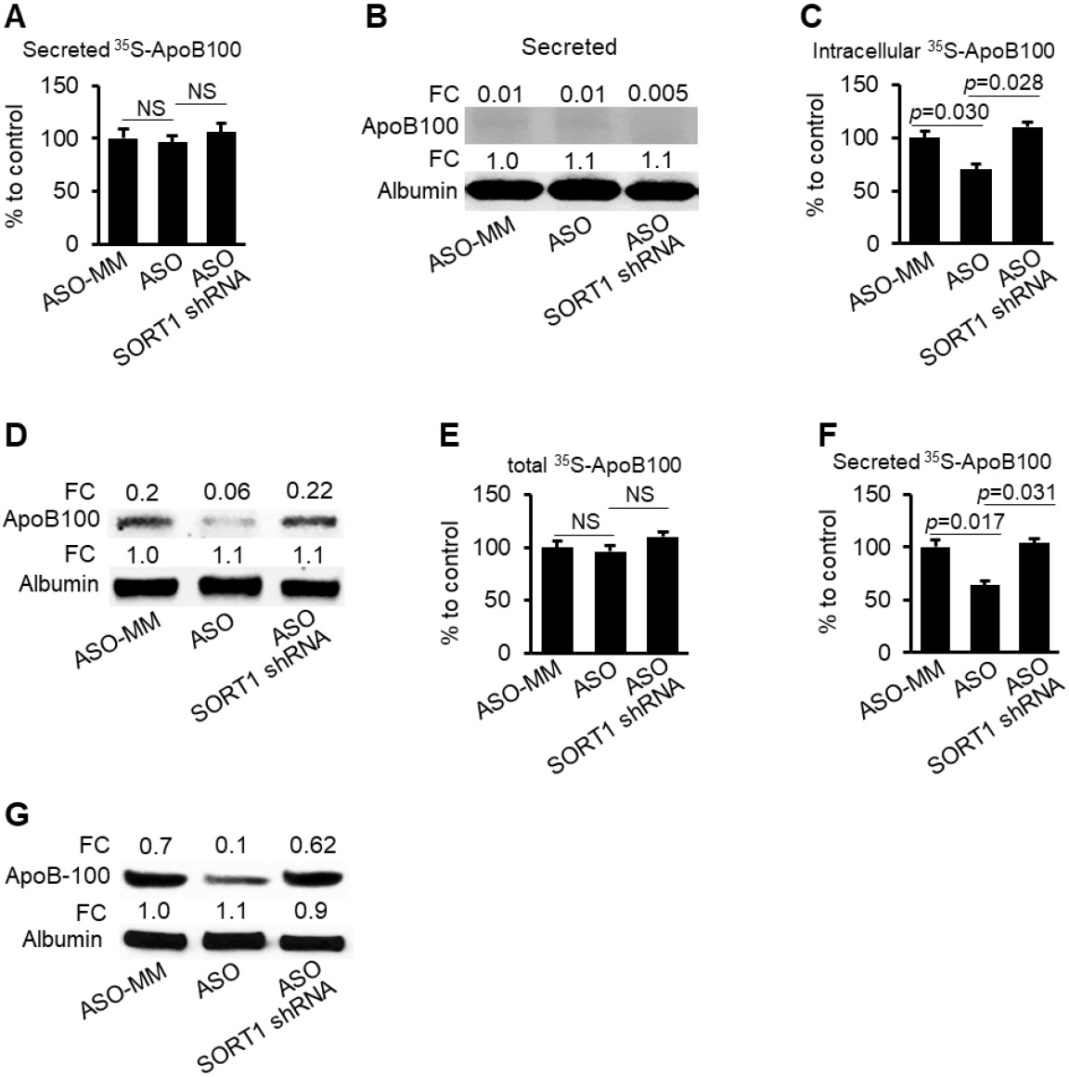
** miR-378a-3p knockdown promoted degradation of ApoB100 and impaired secretion of ApoB100 by increasing *SORT1*.** (**A**-**B**) CP-10447 inhibited secretion of ApoB100, which was reflected by almost undetectable ^35^S-ApoB100 in the medium among three groups of HepG2-miR cells. HepG2-miR cells were transfected with scramble (control), miR-378a-3p-ASO or a combination of miR-378a-3p-ASO and *SORT1* shRNA. 48 hours post-transfection, cells were starved for one hour in cysteine and methionine free medium and then labeled with ^35^S-methionine/cysteine for 3 hours in the presence of CP-10447. (**C**-**D**) miR-378a-3p knockdown reduced intracellular ^35^S-ApoB100, while additional treatment of *SORT1* shRNA offset the effect of miR-378a-3p-ASO. (**E**) No significant change was observed in total ^35^S-ApoB100 (intracellular and medium) among three groups of HepG2-miR cells transfected with scramble (control), miR-378a-3p-ASO or a combination of miR-378a-3p-ASO and *SORT1* shRNA. HepG2-miR cells were transfected with scramble (control), miR-378a-3p-ASO or a combination of miR-378a-3p-ASO and *SORT1* shRNA. 48 hour post transfection, cells were starved for one hour in cysteine and methionine free median and then labeled with ^35^S-methionine/cysteine for 3 hours in the presence of MG132. (**F-G**) miR-378a-3p knockdown reduced secreted ^35^S-ApoB100 in the medium of HepG2-miR cells; while additional treatment of *SORT1* shRNA counteracted the effect of miR-378a-3p-ASO. Data represent mean ± SEM. **p < 0.01 (AVONA test).

To test whether *SORT1* mediates the effects of miR-378a-3p on degradation and secretion of ApoB100, three groups of HepG2-miR cells maintained in the medium containing CP-10447 or MG132 were transfected with scramble (control), miR-378a-3p-ASO or a combination of miR-378a-3p-ASO and *SORT1* shRNA. miR-378a-3p-ASO treatment resulted in decreased miR-378a-3p and increased *SORT1* in HepG2-miR cells (Figure S5). Phenotypically, miR-378a-3p knockdown promoted ApoB100 degradation in HepG2-miR cells treated with CP- 10447 (Figure 5A-D) and inhibited secretion of ApoB100 in HepG2-miR cells treated with MG132 (Figure 5E-G), while additional treatment of *SORT1* shRNA offset the inhibitory effects of miR-378a-3p- ASO on stability and secretion of ApoB100 (Figure 5). These findings indicate that SORT1 is required for miR-378a-3p to increase stability and secretion of ApoB100.

### miR-378a-3p promotes hepatic secretion of VLDL

**Figure 6:**
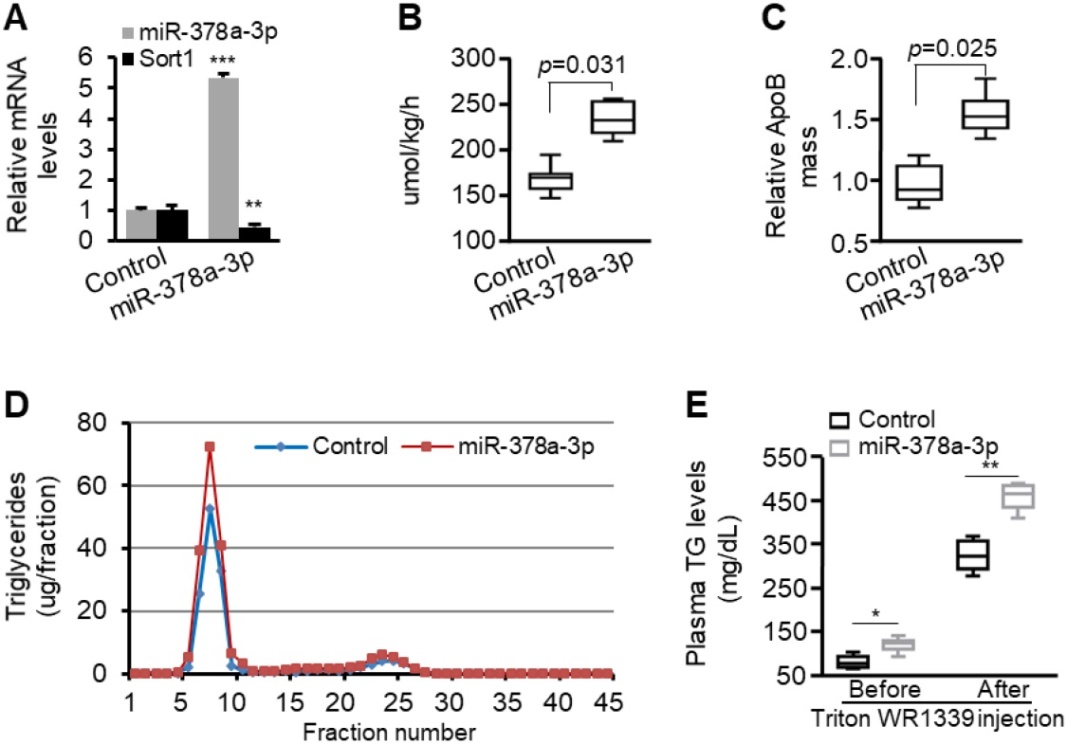
** miR-378a-3p increased the rate of VLDL production in mice.** (**A**) Levels of miR-378a-3p and *Sort1* mRNA in livers of mice treated with MC-*TTR*-miR-378a (*n*=9) or MC-*TTR*-miR-378a-MM (control, *n*=9). (**B**) Increased rate of VLDL production in mice treated with MC-*TTR*-miR-378a. The difference between baseline and 1-hour plasma TG levels was measured after Triton WR1339 treatment. (**C**) Increased ^35^S-methionine/cysteine-labeled ApoB100 in the blood of mice treated MC-*TTR*-miR-378a. (**D**) Increased VLDL-TG in pooled plasma of mice treated with MC-*TTR*-miR-378a. Plasma lipoprotein triglyceride distribution was determined by FPLC. (**E**) Increased levels of total plasma triglycerides in mice treated with MC-*TTR*-miR-378a before and after injection of Triton WR1339. Data represent mean ± SEM. *p < 0.05, **p < 0.01, and ***p < 0.001 (Mann-Whitney test).

*In vitro* studies confirmed that miR-378a-3p enhances stability and secretion of ApoB100. We, therefore, hypothesized that miR-378a-3p should increase production and secretion of VLDL *in vivo*. To test our hypothesis, eight-week-old C57BL/6 mice were injected with either MC-*TTR*-miR-378a or MC-*TTR*-miR-378a-MM (control) to achieve *Sort1* knockdown. Eight weeks post-injection, mice were fasted for 4 hours and the rate of hepatic VLDL secretion was measured by injecting Triton WR1339 to inhibit lipolysis (thus all VLDL made and secreted by liver remains in circulation in VLDL) and ^35^S-methionine/cysteine to label proteins. MC-*TTR*-miR-378a treatment reduced hepatic *Sort1* mRNA by 54% (Figure 6A). Before Triton WR1339 injection, liver-specific expression of miR-378a-3p led to increased TG, insignificant change in plasma cholesterol, reduced HDL cholesterol and increased VLDL/LDL cholesterol (Figure S6). One hour post-injection of Triton WR1339, the VLDL-TG production rate was significantly increased in miR-378a-3p-treated mice compared with control mice (Figure 6B). The secretion rate of ApoB100 was also increased in miR-378a-3p-treated mice (Figure 6C). FPLC analysis revealed that the increase in plasma TG after Triton WR1339 was exclusively in the VLDL fraction (Figure 6D). Liver-specific expression of miR-378a-3p significantly facilitated hepatic secretion of VLDL, which was reflected by increased levels of TG in VLDL fraction after Triton WR1339 injection (Figure 6E). These data indicate that miR-378a-3p is able to increase production and secretion of VLDL.

### E2F1 activates transcription of miR-378a-3p

**Figure 7:**
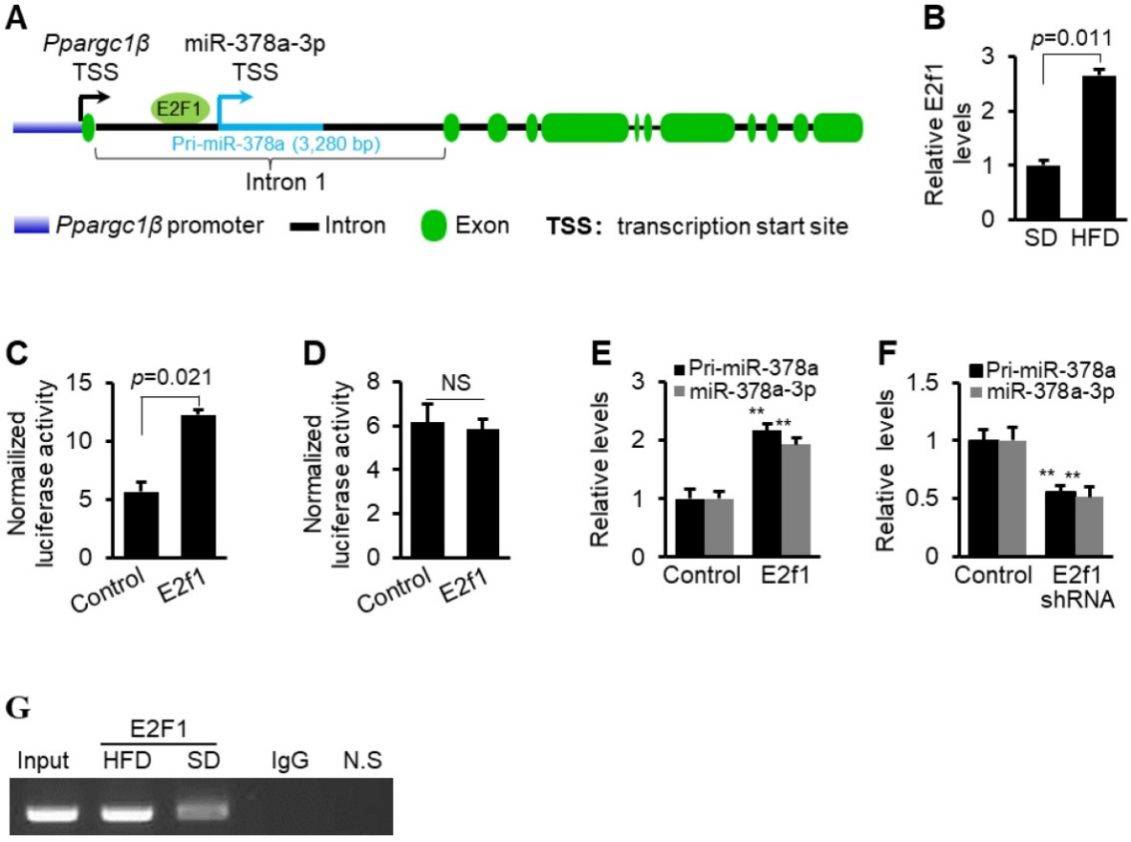
** E2F1 activated transcription of miR-378a-3p. (A)** Schematic representation of E2F1 binding site within the promoter of miR-378a-3p. (**B**) Increased mRNA levels of *E2f1* in livers of HFD-treated mice (*n*=6) versus SD-treated mice (*n*=6). (**C**) Luciferase activities of miR-378a-3p promoter in Hepa1-6 cells transfected with miR-378a-3p promoter construct and *E2f1* expression vector or empty vector (control). (**D**) No change was observed in luciferase activity of miR-378a-3p promoter with the mutated E2F1 binding site after *E2f1* overexpression. (**E**) Increased levels of mature miR-378a-3p and pri-miR-378a in Hepa1-6 cells transfected with *E2f1* expression vector or empty vector (control). (**F**) Reduced levels of mature miR-378a-3p and pri-miR-378a in Hepa1-6 cells transfected with MC-*TTR*-E2f1shRNA or empty vector (control). (**G**) *In vivo* ChIP assays were performed using genomic DNA isolated from livers of mice treated with SD and HFD; and the binding of E2F1 to the endogenous promoter of miR-378a-3p was detected using specific an E2F1 antibody. N.S: non-specific control using primers ∼10 kb downstream of miR-378a-3p promoter which served as negative control. Data represent mean ± SEM. **p < 0.01 (Student* t* test).

Regulation of miRNA biogenesis is controlled at the transcription and/or maturation of miRNAs 40-43. Elucidation of biogenesis mechanism of elevated miR-378a-3p is important to provide a comprehensive explanation on the role of miR-378a-3p in hyperlipidemia. We, therefore, predicted potential binding sites for transcription factors within the promoter of miR-378a-3p 44, and identified a binding site for E2F1 (Figure 7A and Figure S7). E2F1 is an oncogenic transcription factor that has increased expression in fatty livers 23 (Figure 7B). We next investigated whether E2F1 is able to bind to the miR-378a-3p promoter and activate its transcription. The action of E2F1 on the promoter of miR-378a-3p was examined by luciferase assay. Overexpression of *E2f1* significantly increased activity of miR-378a promoter (Figure 7C). The mutation within the E2F1 binding motif impaired the ability of E2F1 to activate transcription of the miR-378a promoter (Figure 7D), indicating that this binding site is essential for increased expression of miR-378a-3p. Furthermore, both mature miR-378a-3p and primary transcript of miR-378a (pri-miR-378a) were significantly increased after *E2f1* overexpression (Figure 7E), while *E2f1* knockdown led to an opposite effect (Figure 7F). These results indicated that miR-378a-3p biogenesis was activated at the transcription level by E2F1.

**Figure 8:**
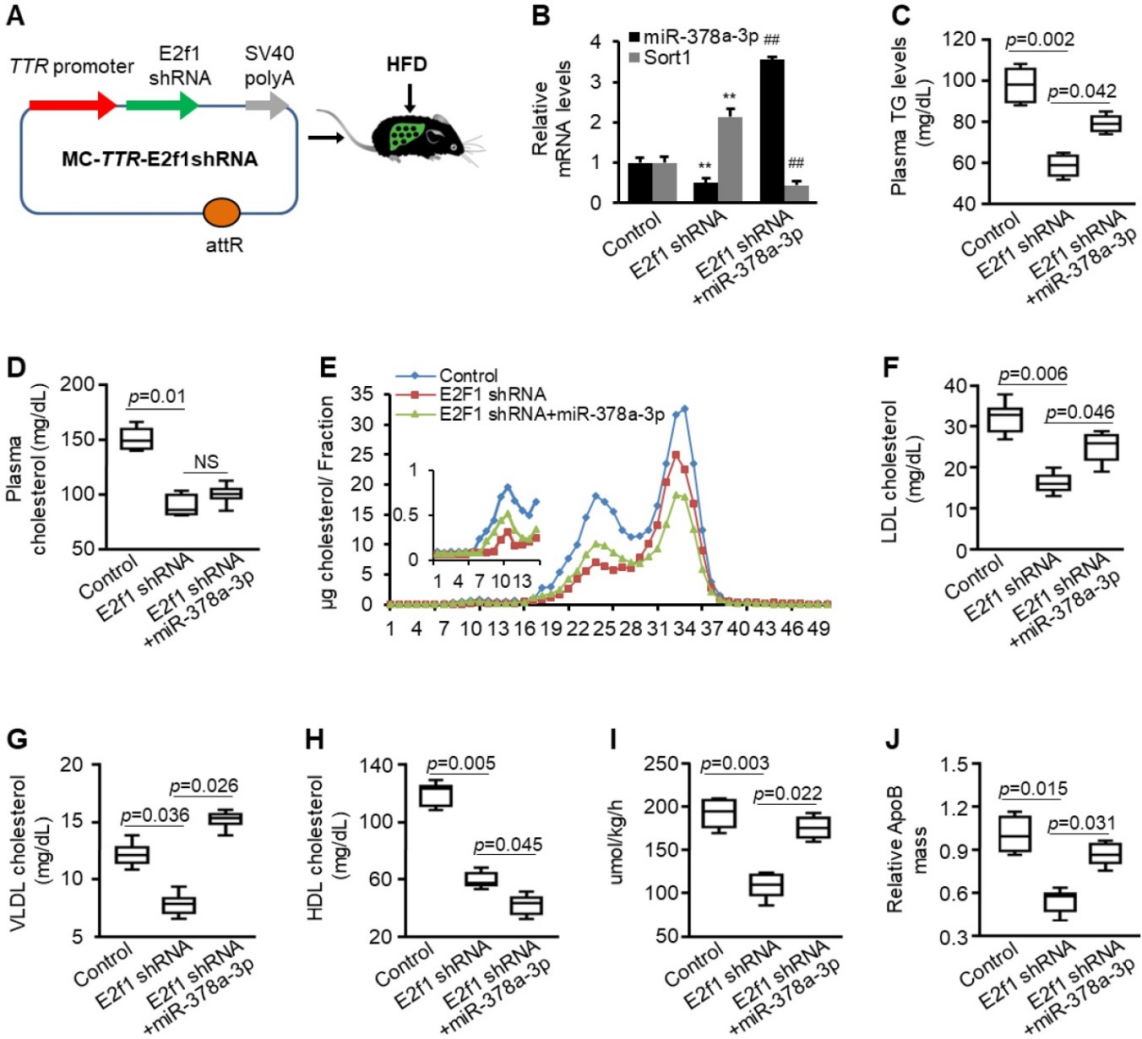
***E2f1* knockdown impaired secretion of VLDL and re-introduction of miR-378a-3p partially offset the effect of *E2f1* knockdown.** (**A**) Diagram of hepatic-specific *E2f1* shRNA expression vector. (**B**) Levels of miR-378a-3p and *Sort1* mRNA in livers of three groups of HFD-treated mice injected with MC-*TTR*-miR-378a-MM (control, *n*=9), MC-*TTR*-E2f1shRNA (*n*=9), or a combination of MC-*TTR*-E2f1shRNA and MC-*TTR*-miR-378a (*n*=9). (**C**) *E2f1* knockdown reduced levels of total plasma triglyceride, while additional treatment of miR-378a-3p partially restored decreased plasma triglyceride (pooled plasma). Blood was collected before Triton WR1339 injection. (**D**) *E2f1* knockdown reduced plasma cholesterol, while additional treatment of miR-378a-3p failed to recover reduced plasma cholesterol (pooled plasma). (**E-H**) FPLC analysis of pooled plasma revealing that *E2f1* knockdown reduced levels of VLDL, LDL and HDL cholesterol. In contrast, re-introduction of miR-378a-3p partially restored reduced LDL/VLDL cholesterol but further reduced HDL cholesterol. (**I**) *E2f1* knockdown impaired VLDL production and miR-378a-3p recovered VLDL production that was impaired by *E2f1* knockdown. (**J**)* E2f1* knockdown reduced ^35^S-methionine/cysteine-labeled ApoB100; and additional treatment of miR-378a-3p offset the effect of *E2f1* shRNA. Data represent mean ± SEM. **p < 0.01 (ANOVA test).

To examine whether E2F1 physically interacts with the promoter of miR-378a-3p, we performed chromatin immunoprecipitation (ChIP). DNA fragment containing E2F1 binding site within the promoter of miR-378a-3p was immune-precipitated from genomic DNA from livers of mice treated with the SD and HFD by an E2F1 antibody (Figure 7G). In addition, HFD treatment led to more immune precipitation of DNA containing E2F1 binding site, suggesting that E2F1 can directly interact with its corresponding binding site within the miR-378a-3p promoter and HFD-induced E2F1 led to increased miR-378a-3p. To examine whether the interaction between E2F1 and miR-378a-3p exists *in vivo*, we constructed a mini-circle vector containing *E2f1* shRNA driven by liver-specific *TTR* promoter (Figure 8A). This vector was referred as to MC-*TTR*-E2f1shRNA. As expected, injection of MC-*TTR*-E2f1shRNA into livers of mice impaired expression of miR-378a-3p (Figure 8B). Together, E2F1 can physically bind to the promoter of miR-378a-3p and activate its transcription.

### *E2f1* knockdown partially simulates the function of miR-378a-3p-ASO in inhibiting hepatic secretion of VLDL

We hypothesized that E2F1 contributes to high levels of circulating LDL cholesterol and TG by activating miR-378a-3p-SORT1 axis. To test this hypothesis, eight-week-old mice were treated with HFD for 8 weeks. Mice were then allocated into three groups. Group I was treated with injected with *E2f1* shRNA; Group II was treated with a combination of *E2f1* shRNA and MC-*TTR*-miR-378a; and Group III received MC-*TTR*-miR-378a-MM (control). Such a design allows us to determine whether E2F1 can, through activating miR-378a-3p biogenesis, increase hepatic secretion of VLDL. After 8 weeks of injection, mice were fasted for 4 hour and blood were collected. Mice were then injected with Triton WR1339 and ^35^S-methionine/cysteine to inhibit lipolysis and label ApoB100.

*E2f1* knockdown led to reduced miR-378a-3p and increased expression of *Sort1*, while additional treatment of MC-*TTR*-miR-378a recovered expression of miR-378a-3p and repressed elevated *Sort1* (Figure 8B). Consistent with the role of E2F1 in activating lipogenesis, *E2f1* knockdown impaired expression of *Srebp1c, Scd1*, and *Fasn* and reduced hepatosteatosis, while additional treatment of miR-378a-3p failed to recover expression of these genes but still partially recovered hepatic lipid accumulation (Figure S8). This finding is consistent with our recent report that miR-378a-3p promotes hepatosteatosis by impairing FAO 21. Before Triton WR1339 injection, both plasma cholesterol and TG were reduced after *E2f1* knockdown; in contrast, additional treatment of miR-378a-3p partially recovered levels of plasma TG but failed to restore reduced plasma cholesterol (Figure 8C-D). This finding further indicated that miR-378a-3p had no effect on levels of total plasma cholesterol. As revealed by FPLC, *E2f1* knockdown led to reduced levels of VLDL, LDL and HDL cholesterol; while additional treatment of miR-378a-3p offset the inhibitory effects of *E2f1* shRNA on VLDL/LDL cholesterol but further reduced HDL cholesterol (Figure 8E-H).

We further analyzed blood from mice after injection of Triton WR1339. As expected, *E2f1* knockdown reduced the rate of VLDL production (Figure 8I). Consistent with this finding, the secretion of ApoB100 was also decreased after *E2f1* knockdown (Figure 8J). To determine whether miR-378 mediates the promoting effect of E2F1 on VLDL secretion, we compared mice treated with *E2f1* shRNA and a combination of *E2f1* shRNA and miR-378a-3p. As expected, re-introduction of miR-378a-3p recovered the rate of VLDL production and ApoB100 secretion that were repressed by *E2f1* knockdown (Figure 8I-J). Together, these findings indicated that E2F1-miR-378a-3p-SORT1-ApoB100 axis promotes high levels of plasma TG and LDL/VLDL cholesterol by increasing VLDL production.

## Discussion

Approximately 50% adults have hypercholesterolemia and hypertriglyceridemia 45. The main contributing factor for these two disorders is elevated levels of circulating LDL cholesterol and TG, which ultimately results in the formation of atherosclerosis and plaques that block coronary arteries 46. Statins, as a standard-of-care approach for hyperlipidemia, can only reduce the risk of coronary artery disease by 20-40% 7. For patients with heart failure or other end-stage diseases, statins offer very limited benefits 47, indicating the importance of developing new therapeutic approaches against hyperlipidemia and CVD. miR-378a-3p is such a molecule that exhibits the great potential as a novel therapeutic target for both hypertriglyceridemia and hypercholesterolemia. By administering miR-378a-3p to HFD-treated mice, we have shown that miR-378a-3p is a robust promoter of hepatic secretion of VLDL and aggravates the pathogenesis of hypertriglyceridemia and hypercholesterolemia in laboratory mice. miR-378a-3p knockdown impaired secretion of VLDL and significantly alleviated high levels of circulating VLDL/LDL cholesterol and TG. Due to the important role of ApoB100 in the VLDL production, miR-378a-3p increased stability and secretion of ApoB100. Mechanistically, we identified a previously unrecognized metabolic pathway composed of E2F1, miR-378a-3p, SORT1 and ApoB100 that promotes hepatic secretion of VLDL. In response to HFD challenge, *E2f1* expression is increased 23, which subsequently activates transcription of miR-378a-3p. Increased miR-378a-3p further inhibits expression of *Sort1*, which subsequently promotes hepatic secretion of VLDL and aggravates the development of dyslipidemia. miR-122 is the most abundant hepatic miRNA that plays an important role in inhibiting FAO and promoting *de novo* lipogenesis 48. However, our data showed that no significant change in miR-122 was observed in hepatosteatotic livers, leading us to focus on other most differentially-expressed miRNAs. miR-132 is another significantly elevated miRNA in fatty livers of HFD-treated mice 49. Antagonizing miR-132 leads to reduced hepatosteatosis and hyperlipidemia by regulating multiple targets 49. All these findings suggested that miRNAs, as a group, coordinately maintains lipid homeostasis.

The plasma level of ApoB100 is among the strongest risk factors for coronary artery disease, making the regulation of its production by the liver a significant pursuit. However, knowledge about the regulated production of ApoB100 by the liver is vital to the understanding of hyperlipidemia and to the identification of factors that can be targeted to reduce high levels of atherogenic apoB-lipoproteins 50. Some literatures have reported that ApoB100 production by the liver is regulated primarily by post-translational degradation 51. Manipulation of hepatic *Sort1* lowered levels of plasma LDL cholesterol and TG by inhibiting hepatic apoB100/ VLDL production in mice 17, 52. Furthermore, GWAS established that a non-coding polymorphism within *Sort1* is closely related to CVD in humans, and miR-378a-3p and its target *Sort1* are conserved between human and mouse 17, suggesting that the novel findings in this study can be applied to humans. In contrast, another report showed that sortilin overexpression stimulates hepatic release of lipoproteins and increases plasma LDL levels 53. A reasonable explanation for these contradictory findings is that *Sort1* deficiency studies were done in a total body knockout moue, whereas the GWAS, overexpression and knockdown studies are involved liver-specific manipulations exclusively 53. Further studies are needed to address this controversy. Another function of SORT1 is to increase LDL catabolism 52, suggesting that miR-378a-3p has potential to impair LDL catabolism. To test this speculation, we performed fluorescently-labeled LDL uptake assay in hepatocytes. Both gain- and loss-of functions of miR-378a-3p revealed that miR-378a-3p had no effect on LDL uptake in hepatocytes (Figure S9). The potential explanation for this observation is that miR-378-3p can target other genes that have opposite function compared to sortilin. As discussed above, more controversial results regarding sortilin were observed. Further studies are needed to elucidate the detailed function of SORT1 in lipoprotein metabolism.

Another major goal of this study was to delineate the molecular mechanisms of activated miR-378a-3p biogenesis. Although dysregulated miRNAs are involved in the pathogenesis of hyperlipidemia 15, 48, most of the studies focus on targets of miRNAs. Mechanisms of miRNA deregulation in hyperlipidemia and CVD are rarely described. Notably, different types of diseases are highly associated. For example, the association of hyperlipidemia with cancer began with early observations of an accumulation of cholesterol in tumors 54. It is very possible that some transcription factors modulate the pathogenesis of these highly-associated diseases by modulating expression of a certain miRNA. In this study, we observed that the oncogenic E2F1 is a transcription activator of miR-378a-3p. E2F1 is a promoter of liver cancer by controlling cell cycle 55. A recent literature reported that E2F1 mediates sustained lipogenesis and contributes to hepatosteatosis 23. However, its role in regulating lipoprotein metabolism is unknown. This study presented important evidence to support that E2F1 also serves as a promoter of hepatic secretion of VLDL and hyperlipidemia by modulating miR-378a-3p-SORT1 axis. Identification of this novel pathway provided an important target to design a therapeutic approach to simultaneously treat highly-associated liver cancer and hyperlipidemia.

## Conclusion

We concluded that the E2F1-miR-378a-3p-SORT1 axis is essential in the control of levels of circulating VLDL/LDL cholesterol and TG. Upon activation, this axis maintains activation of miR- 378a-3p biogenesis, suppression of *Sort1*, increased stabilization and secretion of ApoB100, which subsequently facilitates hepatic secretion of VLDL and aggravates the pathogenesis of hyperlipidemia and hypolipoproteinemia. The insights obtained from this study further advance our understanding of the physiological roles of miR-378a-3p and regulation of miR-378a biogenesis in lipoprotein metabolism, in addition to developing a potential therapeutic agent against hyperlipidemia.

## Supplementary Material

Supplementary materials and methods, figures.

## Abbreviations

ApoB100: apolipoprotein B 100
ChIP: chromatin immunoprecipitation
CVD: cardiovascular disease
FPLC: fast phase liquid chromatography
GWAS: genome wide association study
HFD: high fat diet
HITS-CLIP: high-throughput sequencing of RNAs isolated by crosslinking immunoprecipitation
LDL: low-density lipoprotein
miR-ASO: anti-sense oligonucleotide of miRNAs
NAFLD: non-alcoholic fatty liver disease
PCSK: proprotein convertase subtilisin/ kexin family
qRT-PCR: quantitative real-time polymerase chain reaction
shRNA: short-hairpin RNA
TTR: transthyretin gene
VLDL: very low- density lipoprotein
and 3' UTR: 3' untranslated region
