# Supplementary material for: Activation of microRNA-378a-3p biogenesis promotes hepatic secretion of VLDL and hyperlipidemia by modulating ApoB100-Sortilin1 axis
Source: Theranostics. 2020 Mar 4;10(9):3952–66. doi: 10.7150/thno.39578 (PMC7086368; doi:10.7150/thno.39578)
Supplement: Supplementary file 1 — Supplementary materials and methods, figures. [file thnov10p3952s1.pdf]

Activation of microRNA-378a-3p biogenesis promotes hepatic secretion of VLDL and hyperlipidemia by modulating ApoB100-Sortilin1 axis

Authors: Tianpeng Zhang[1,] *, Hongtao Shi[2,] *, Ningning Liu[1], Jing Tian[2], Xiaoling Zhao[3], Clifford J. Steer[1], Qinghua Han[2, #] and Guisheng Song[1, #]

Affiliations:

[1]Department of Medicine, University of Minnesota Medical School, Minneapolis, Minnesota 55455

[2]Department of Cardiology, the First Hospital of Shanxi Medical University, Taiyuan City, China 030001

[3]MC Lab, South San Francisco, CA 94080

*Tianpeng Zhang and Hongtao Shi equally contributed to this manuscript.

Contract information:

[#]To whom correspondence should be addressed: Guisheng Song, Ph.D., 516 Delaware Street SE, Minneapolis MN 55455, Tel: 1-61-924-9961, E-mail: gsong@umn.edu

Or Qinghua Han, M.D., Department of Cardiology, the First Hospital of Shanxi Medical University, Taiyuan City, China, Email: sygqh@sohu.com

Supplementary Materials and Methods

3'UTR reporter vector construction and luciferase assay

To generate the luciferase reporter vectors, 3' UTR of *Sort1* were amplified by PCR from mouse cDNA, and inserted into the pMiR-Reporter vector (Ambion), referred as pMiR-*Sort1*. Two bases of the binding sites for miR-378 within the 3'UTR of *Sort1* were mutated using QuikChange II Site-Directed Mutagenesis Kit (Agilent Technologies) per the manufacture's instruction, and referred as pMiR-Mu-*Sort1*. 24 hours before transfection, 5×10^4 Hepa1-6 cells were plated per well in a 24-well plate. Then, 200 ng of the luciferase reporter vector and miR-378a-3p mimic (20 nM) as well as 30 ng of β-*gal* plasmid pSV-β-Galactosidase Control Vector (Promega) were transfected into Hepa1-6 cells using Lipofectamine 2000 (Invitrogen). Scrambled control (Dharmacon) was used as the control for miR-378a-3p mimic. After 24 hours of transfection, luciferase and β-galactosidase assays were done using the Luciferase Assay System and Beta-Glo® Assay System (Promega). Luciferase activities were normalized to galactosidase activities; wells were transfected in triplicate; and each well was assayed in triplicate.

Chromatin immunoprecipitation (ChIP) Assay

Approximately 130 mg of livers from mice treated with SD or HFD was used for ChIP assay. ChIP assay was performed using the ChIP Assay Kit (Abcam, ab500) based on the manufacture's protocol. E2F1 antibody was purchased from Abcam (ab4070). The binding region was detected in PCR reactions. A 10 kb region downstream from the binding site was used as a negative control and chromatin solution was reserved for input control. Primers flanking the binding site of E2F1 within the murine miR-378a-3p promoter were: forward, 5′-CTAGACTAGGAGCAGGAAAAGC -3′ and reverse, 5′- GCAGCACAAGGCACGTGGAAG -3′. The negative control primers were: forward, 5′-

CTTCTATATGAAGAGACAGAGTAC -3′ and reverse, 5′-TGGAGTCCCTGCTATGTAGAGCCAG -3′.

Reporter vector construction and luciferase assay

To generate the luciferase reporter vector of miR-378a-3p promoter, the promoter of miR-378a-3p was amplified from mouse genomic DNA using PCR, and inserted into pGL3-basic vector (Promega), referred as pGL3-miR-378. Two bases of the binding site for E2F1 within the promoter of miR-378 were mutated using QuikChange II Site-Directed Mutagenesis Kit (Agilent Technologies) per the manufacture's instruction, and referred as pGL3-Mu-miR-378. 24 hours before transfection, 5×10^4 Hepa1-6 cells were plated per well in a 24-well plate. Then, 200 ng of the luciferase reporter vector and *E2f1* expression vector (pCDNA3.1-E2F1, Addgene, 200 ng) as well as 30 ng of β-*gal* plasmid pSV-β-Galactosidase were transfected into Hepa1-6 cells using Lipofectamine 2000 (Invitrogen). Hepa1-6 cells treated with pGL3-miR-378 and empty vector was used as the control. After 24 hours of transfection, luciferase and β-galactosidase assays were done using the Luciferase Assay System and Beta-Glo® Assay System (Promega). Luciferase activities were normalized to galactosidase activities; wells were transfected in triplicate; and each well was assayed in triplicate.

miRNA transfection and gene expression

5×10^4 of Hepa1-6 cells were seeded in a 24-well plate and allowed to adhere overnight. To determine the effects of miR-378a-3p overexpression and knockdown on gene expression, Hepa1-6 cells cultured in the DMEM with 10% FBS were transfected with miR-378a-3p mimic (20 nM) or miR-378a-3p-ASO using Lipofectamine 3000. Hepa1-6 cells transfected with scramble control served as the control. 24 hours after transfection, cells were washed using cold PBS and the total RNA were isolated for gene expression analysis.

Histological analysis

Liver samples were embedded in Tissue-Tek OCT embedding compound, and frozen on dry ice. 8 μm-thick sections were cut with a Leica CM3050 S cryostat, air-dried, and fixed in 10% formalin. After washing, sections were stained with an Oil-Red-O (Sigma-Aldrich)/60% isopropanol solution (Fisher Scientific). Briefly, sections were rinsed with 60% isopropanol and stained for 20 min with prepared Oil Red O solution (0.5% in isopropanol followed by dilution to 60% with distilled water and filtered).

RNA isolation and quantitative reverse transcription-PCR (qRT-PCR)

Total RNA was isolated with miRNeasy Mini Kit (Qiagen). To assess gene expression, 1 μg RNA was used for cDNA synthesis with Superscript III reverse transcription reagent (Invitrogen). PCR amplification was performed at 50°C for 2 minutes and 95°C for 10 minutes, followed by 40 cycles at 95°C for 15 seconds and 60°C for 1 minute in a 7900 real time-PCR system with SYBR green (Applied Biosystems). For each sample, we analyzed β-actin, GAPDH or 18S rRNA expression to normalize target gene expression. Primers for qRT-PCR were designed with Primer Express software (Applied Biosystems).

To determine levels of miRNA expression, 10 ng RNA were used for miRNA-specific cDNA synthesis with the TaqMan MicroRNA Reverse Transcription Kit and Taqman MicroRNA Assays (all Applied Biosystems). PCR amplification was performed at 95°C for 10 minutes, followed by 40 cycles at 95°C for 15 seconds and 60°C for 1 minute in a 7900 real time-PCR system (Applied Biosystems). The small RNA Sno202 and RNU6 were used to normalize target miRNA expression. Relative changes in gene and miRNA expression were determined using the $2^{-\Delta\Delta Ct}$ method [1].

Hepatic lipid analysis

Mouse liver (100 mg) was placed in 1 mL chloroform/methanol (2:1) mixture and incubated on mice for 10 minutes before homogenization. Lipids were extracted from liver homogenates through room

temperature orbital shaking (2 hours) followed by centrifugation (5000 RPM for 5 minutes). Supernatants were collected and washed with 0.4 mL chloroform/methanol (2:1) mixture by centrifugation at 5000 RPM for 20 minutes (room temperature). New supernatants were washed with 0.2 volume of 0.9% NaCl. After centrifuging for 5 minutes at 5000 RPM, supernatants were removed and lower-phase was dried at 42°C. Dried lipids were re-suspended in 2% Triton X-100. Liver triglycerides were quantified via a colorimetric assay using a triglyceride assay kit from Roche Diagnostics according to the manufacturer's protocols.

Blood lipid analysis

Blood was collected into tubes from cardiac puncture of C57BL/6 mice. Serum was separated by centrifugation (3000 x RPM for 20 min at 4 °C and triglyceride (mg/dL, Roche Diagnostics) was quantified enzymatically. Serum chemistry was carried out by the Pathology Laboratory of the University of Minnesota.

MTP activity assay

MTP activity in liver homogenate was measured using a MTP Activity Assay Kit (Sigma) according to the manufacturer's protocol.

LDL Uptake Assay

HepG2 or Hepa1-6 cells were transfected with either miR-378a-3p mimic or scramble control in a 96-well plate. 48 h post transfection, cell culture medium was removed from the 96-well plate, and cells were incubated overnight at 37 °C in serum-free EMEM medium containing human LDL conjugated to DyLight™ 549, a fluorescent probe for detection of LDL uptake (LDL Uptake Cell-Based Assay kit; Cayman). The degree of LDL uptake was examined in an Evos Digital Inverted Fluorescence Microscope (AMG, Fisher Scientific) with filters for excitation at 540 nm and emission at 570 nm. The

intensity of fluorescence was quantified at 540/570 nm excitation/emission using a Synergy MX plate reader (Biotek, Winooski, VT).

Western blot analysis

Western blot was performed following standard procedures and analyzed by LICOR-Odyssey infra-red scanner. Primary antibodies of SORT1 (Abcam, Cat. No.: ab16640), E2F1 (Abcam, Cat. No.: ab218527), ApoB100 (Calbiochem, Catalog No. 178467), and albumin (Midland Bioproducts Corp., Catalog No. 71907), ApoB48 (Abcam, Cat. No.: ab20737) and Transferrin (Abcam, Cat. No.: ab82411) were purchased.

References

[1] Schmittgen TD, Livak KJ. Analyzing real-time PCR data by the comparative C(T) method. Nature Protocol 2008;3:1101-1108.

Supplementary Figures

Supplementary Figure 1

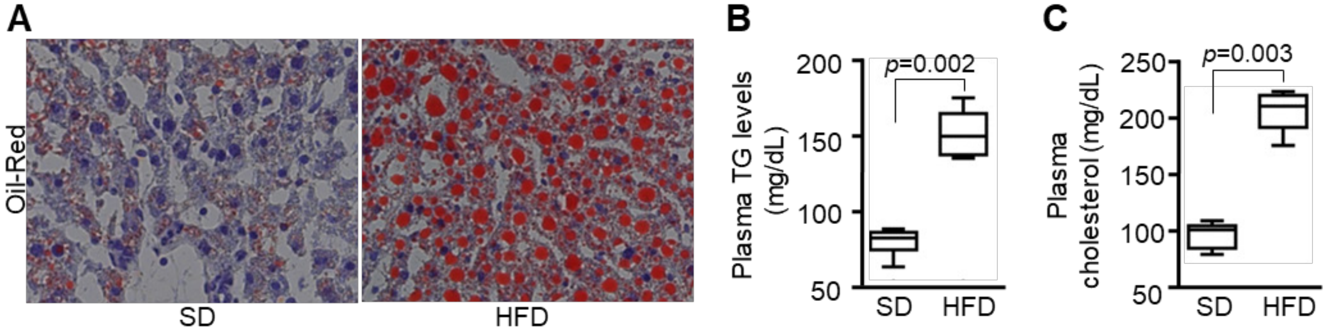

Supplementary Figure 1 HFD treatment induced hepatosteatosis and hyperlipidemia. (A) Oil-Red staining of livers from mice treated with either standard diet (SD) (*n*=6) or high fat diet (HFD) (*n*=6) for 8 weeks. (**B**) Increased levels of plasma triglyceride in mice treated with HFD compared to mice treated with SD. (**C**) Increased levels of total plasma cholesterol in mice after 8 weeks of HFD treatment. Data represent mean ± SEM (Mann-Whitney test). *P* values are indicated.

Supplementary Figure 2

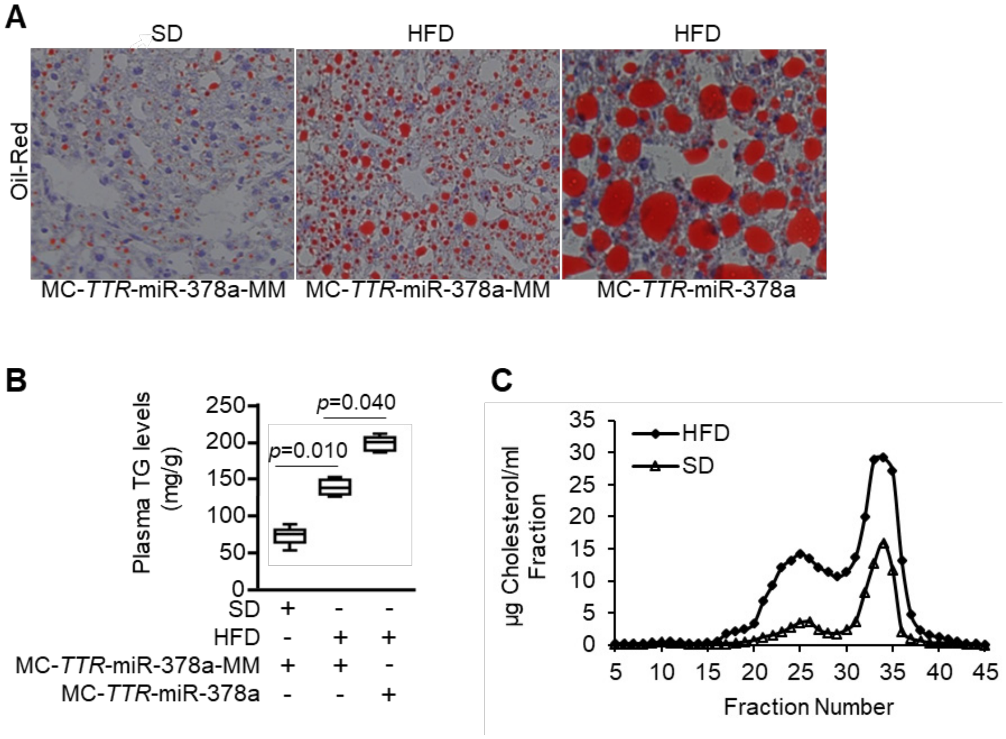

Supplementary Figure 2 Liver-specific expression of miR-378a-3p promoted hepatosteatosis. (**A**) HFD treatment induced hepatic lipid accumulation, while MC-*TTR*-miR-378a treatment further promoted hepatic lipid accumulation. Oil-Red staining of livers from three groups of mice treated with SD and MC-*TTR*-miR-378a-MM (n=9), HFD and MC-*TTR*-miR-378a-MM (n=9), and MC-*TTR*-miR-378a (n=9) for 8 weeks. (**B**) Levels of plasma triglyceride in three groups of mice. (**C**) FPLC profile of pooled plasma from mice treated with SD or HFD. Data represent mean ± SEM (Student t test). P values are indicated.

Supplementary Figure 3

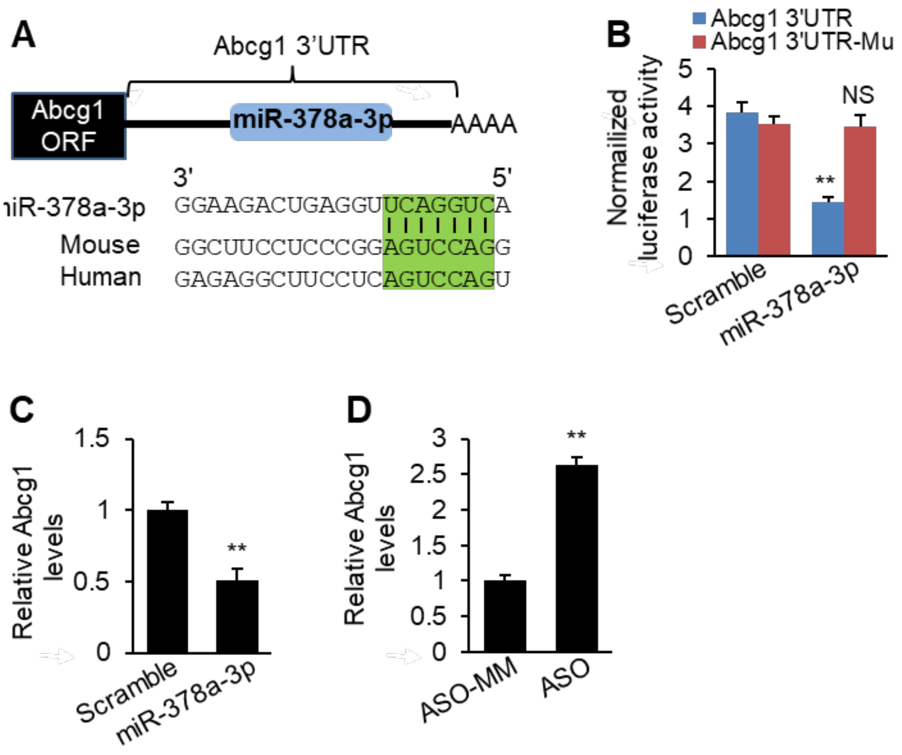

Supplementary Figure 3 *Abcg1* is a direct target of miR-378a-3p. (**A**) Graphic representation of the conserved miR-378a-3p binding motifs within the 3'UTR of *Abcg1*. Complementary sequences to the seed regions of miR-378a-3p within the 3'UTRs are conserved between human and mouse (highlighted in green). (**B**) Luciferase activity of the luciferase reporter constructs containing either wild-type or mutated 3'UTR of murine *Abcg1* after miR-378a-3p mimics treatment. Hepa1-6 cells treated with scramble served as control. NS: no significance. (**C**) Reduced mRNA levels of *Abcg1* in Hepa1-6 cells transfected with miR-378a-3p mimics. Hepa1-6 cells transfected with scramble served as the control. **(D**) Increased mRNA levels of *Abcg1* in Hepa1-6 cells transfected with miR-378a-3p-ASO. Hepa1-6 cells received scramble served as the control. Data represents mean ± SEM. **$p < 0.01$ (Student t test)

Supplementary Figure 4

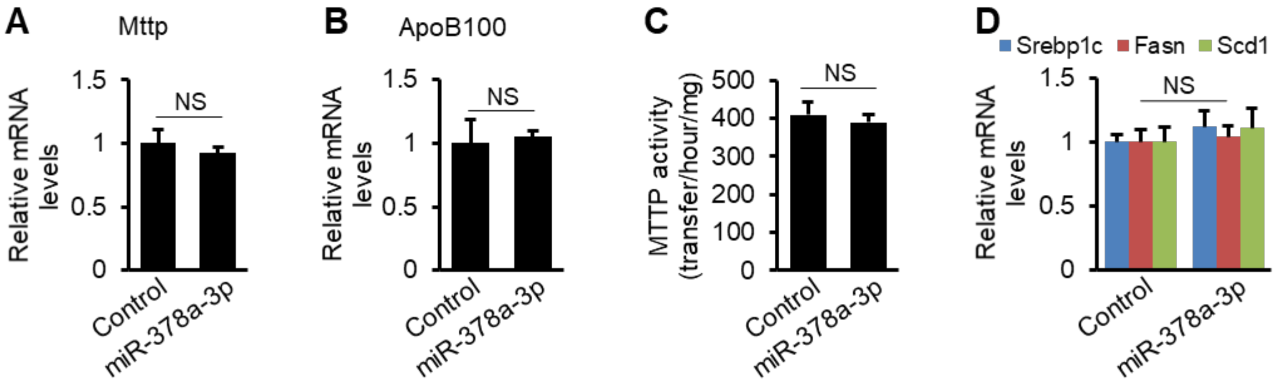

Supplementary Figure 4 The effect of miR-378a-3p on levels of *Mttp* and *ApoB100* as well as lipogenic genes. (**A-B**) No significant change in mRNA levels of *Mttp* and *ApoB100* was observed between two groups of mice treated with MC-*TTR*-miR-378a-MM (control, *n*=9), and MC-*TTR*-miR-378a (*n*=9) for 8 weeks. Mice were kept on normal chow until 8 weeks of age and were then maintained on HFD until 24 weeks of age. At 16 weeks of age, mice were injected with MC-*TTR*-miR-378a-MM or MC-*TTR*-miR-378a weekly for eight weeks. (**C**) No change in MTP activity between two groups of mice. (**D**) No significant change in mRNA levels of *Srebp1c*, *Scd1* and *Fasn* between groups of mice. Data represent mean ± SEM (Student *t* test). *P* values are indicated.

Supplementary Figure 5

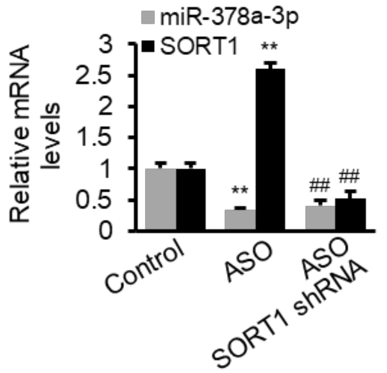

Supplementary Figure 5 miR-378a-3p-ASO treatment led to reduced miR-378a-3p and increased *SORT1*; while additional treatment of *SORT1* shRNA further knocked down increased *SORT1*. HepG2-miR cells were transfected with scramble (control), miR-378a-3p-ASO (ASO) or a combination of miR-378a-3p-ASO and *SORT1* shRNA. 48 hours post transfection, cells were starved for one hour in cysteine and methionine free medium and then labeled with ^{35}S-methionine/cysteine for 3 hours in the presence of CP-10447 or MG132. Data represent mean ± SEM (ANOVA test). $^{**}p < 0.01$ (control versus miR-378a-3p-ASO) and $^{\#\#}p < 0.01$ (miR-378a-3p-ASO versus a combination of miR-378a-3p-ASO and *SORT1* shRNA)

Supplementary Figure 6

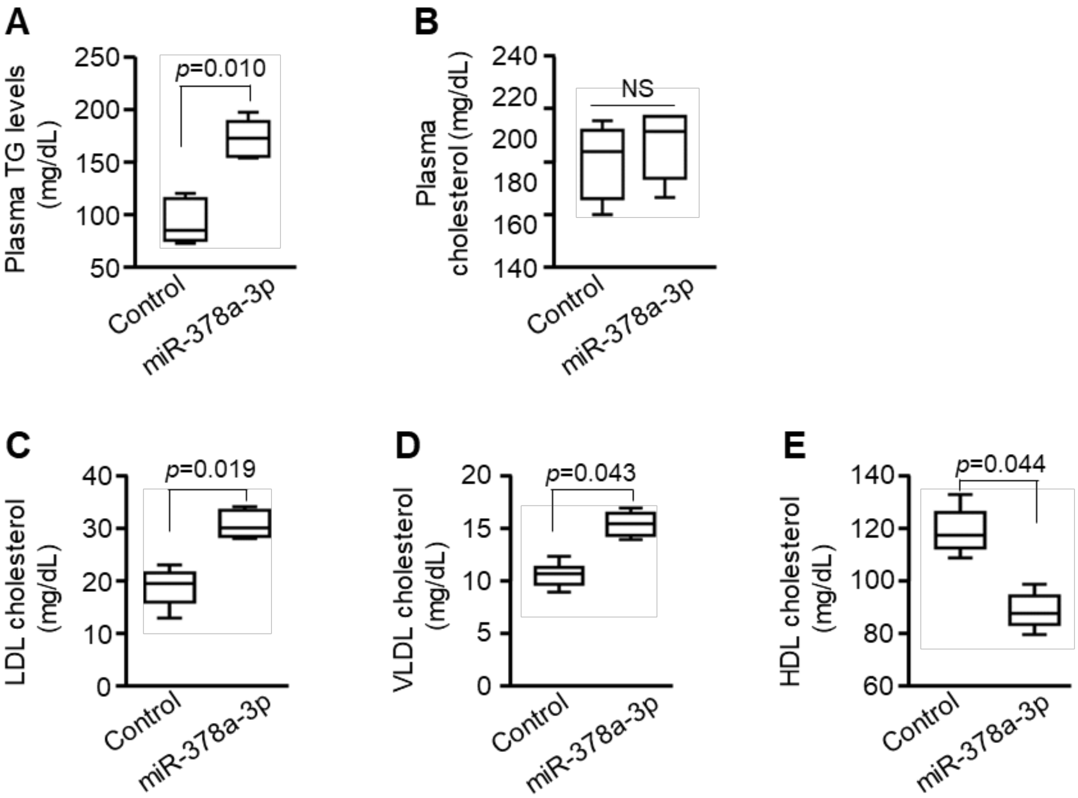

Supplementary Figure 6 Liver-specific expression of miR-378a-3p led to increased plasma TG, insignificant change in plasma cholesterol, increased VLDL/LDL cholesterol and reduced HDL cholesterol. Two groups of mice were injected MC-*TTR*-miR-378a (n=9) or MC-*TTR*-miR-378a-MM (n=9) weekly for 8 weeks. 8 weeks post-injection, Triton WR1339 was injected into both groups of mice. Blood was collected before Triton WR1339 for serum chemistry and FPLC analysis (pooled plasma). Data represent mean ± SEM (Mann-Whitney test). *P* values are indicated.

Supplementary Figure 7 E2F1 binding sites with the miR-378a promoters of mouse and human

Promoter of mouse miR-378a-3p

TAAGGAGGGGGTGCCCGCATTGCTGCGCAGGCACCCGGCTGCTGGCCCGGGCACCGCCACCTGGCTGC
CGGAGCTTTTTTTTTTTCCCCTGCGGGGGGCTGCGAGACCTGGTCCCTGCGCTAGACTAGGAGCAGGAAA
AGCCGGGGGTGCGACCCCAGGCTCGCGCGGCCCCGCCCCCGCAGCCAGCCCGCCTGCCGCAGCCGGG
GGCCCGAGCTCCGCCCTCCACCCGCCCGCCGGGCAGCCTCCTCCCTCCTCCTCGCTCGCTTGCTCGCTC
GCTCGCTCCCTCCCCCGGGCGGGCTCGGCGCTGACTCCGCCGCACGCTGCAGCCGCGGCTGGAAGATG
GCGGGGAACGACTGCGGCGCGCTGCTGGATGAAGAGCTCTCGTCCTTCTTCCTCAACTATCTCTCTGACA
CGCAGGTACTGCCAGCAGGGAACGCAGGCCGGGGACGTGGGTGCAGAACCGCGGGGCTGCAGCCGCAG
CTGCAGAGGGAACGAGCGCAGCGGTGGGCGCACCGGGGTGATCGGGGGGTTCAGGCTGCAGAGCCCCC
CCTTCCACGTGCCTTGTGCTGCGCTCCGTTACAGGCGTAGCGAGCTGCAGGTCGCCGGCGCATGCTGTAG
CATTGGGGGACACAGCCAGGGCCACCTAGAGTCTACTGTCCCCCACTGGCAAAGTTGAGGGGTACTACAA
TTCCTCCCGCAGGTAGAATCCAGCCAGGGTCGGGGGATCCCTAAAGGCTGGCTCTGGGTGTTGTAGGCGC
GTCCTCGGCGCCTGGCACTTGCTGCCGTACTTTCACGTGCACTTGTGGCGGGGCTAAGGGAGGCGATCG
GTAGCCCTGGAGTCCCCTGGTTCTCCAGCATCGGGTACCCCATAGCCCCCCACCTGCTCCGGCAGCGCAG
AGTTTCCTGTCAGTTGCTGGCTAGGGGAATAGGGGTCGGCAGGGCACGTGGACATGCGGGCGGAGATTG
CGTCTTCCAGCTCTCACTAACCGGAGGAGACCCGTTAGGAGCGAGGAAGGCAGGATCCGGGCGGAAGGA
CT**A**

Promoter of human miR-378a-3p

GGGGGCTGCCCGCATTGCTGCGCAGGCATCGGGCTGCTGGCTGGGGCACCGCCACCTGGCTGCCTGCG
CTTTACCCCTTGCTGAGGGCTGCGTGAGCTGGTCGCGGCGCCAGACACGGCGCAGGAAAGTGGGTGAGC
GACCCCCGGCTCCCGCGGGCGCCGCGCGGCCCCGCCCCCGCAGCTAGCGGCCCTGCGGCAGCCGGGG
GCTCGAGCTCCGCCCTCCGCCTCCCGCCGGCCTCACTCCCTCCTCCCTCCTCCCTTGCTCGCTCGCTGGC
TCCCTCCCCCCGGGCCGGCTCGGCGTTGACTCCGCCGCACGCTGCAGCCGCGGCTGGAAGATGGCGGG
GAACGACTGCGGCGCGCTGCTGGACGAAGAGCTCTCCTCCTTCTTCCTCAACTATCTCGCTGACACGCAG
GTACGGCCGGCTGGGGCTGCGGGCCCGGGGCCAGGGGTGCTGAGCTGCGGGGGCCGCAGCTGCAGCC
GCGGAGGCCGGGAGGCAGCGGTGGGAGCCCTGGGGTAACTGGGGGTTCCAGGCTGCAGAGCCCCCCTT
CCAGGCGCCCTGCGATGCGCTCCGTTACCGGGGCAGGGAGCCGGAGGTCTCCCGGCGCGTGCCGGAGC
GCTGGGGGCGCTACGGCCGCTGGGGAGGGTCTAGCCTTGGCCGCTTGGAGTCTGCCACCCCGCGGGCA
AAACTGGGGGGTACCGCGCTTCCTTTGGGAGGTGGAGGCGCGCCACGGTGTGGGGTACCCTCAGGCTGG
CGCTGGGTGCTGGAGGCGCGCCGTCAGCGCCCGGCACTTGCTGCCGTACTTTCACGTGGACTTGTGGCC
CCGGCACGGGTGCATGCCCGGGTCTCCGGAGTCCCCTAGTCCTCCAGGCTGTAGTCCCCAGAGTGCTGTT
GCTGGCCCCCCGCGGCTTTCTACCCGCGCCCGCAGCGCGGAGTTTCCTGCCAGTTGCCGGCTAAAGGCA
TAAGGGTCTGCGGGGCACGTGGACATGCGGGCGGAGACAGCGTCTTCCTGGTTTCGCTATCGGGCGGAG
CCCCCTGGGGGGAAAAGCCAGGCTGGATGGGGGCGGAAGGACT

E2F1 binding site was highlighted in yellow

Supplementary Figure 8

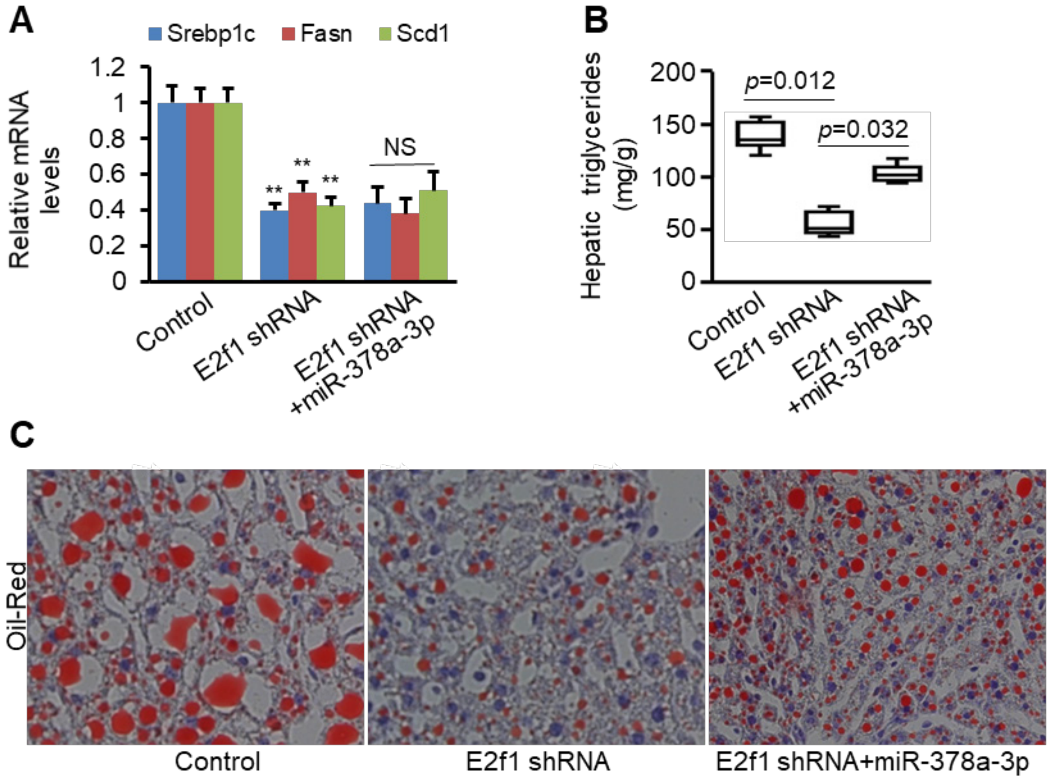

Supplementary Figure 8 *E2f1* knockdown reduced hepatic lipid content, while additional treatment of miR-378a-3p partially recovered hepatic lipid content. (**A**) *E2f1* knockdown reduced mRNA levels of *Srebp1c*, *Scd1* and *Fasn*, while additional treatment of miR-378a-3p failed to recover expression of these genes in livers of three groups of mice. Eight-week-old C57BL/6 mice were kept on HFD. At 16 weeks of age, three groups of mice were injected with MC-*TTR*-miR-378a-MM (control), MC-*TTR*-E2f1shRNA (n=8) or a combination of MC-*TTR*-E2f1shRNA and MC-*TTR*-miR-378a (n=8) weekly for eight weeks. 8 weeks post injection, Triton WR1339 was injected into three groups of mice. (**B**) Hepatic lipid content in three groups of mice. (**C**) Oil-Red staining of livers of three groups of mice. Data represent mean ± SEM. **p<0.01 and NS: no significance (Mann-Whitney test)

Supplementary Figure 9

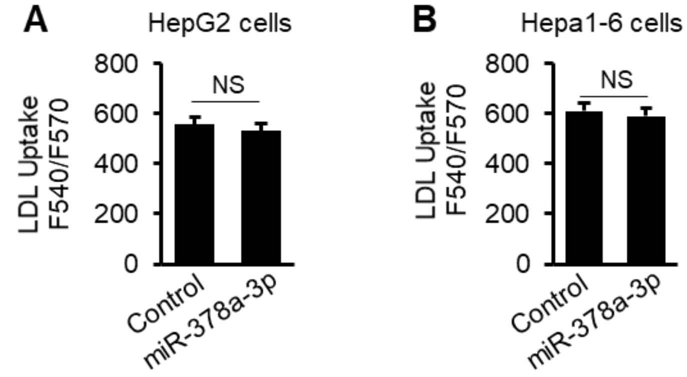

Supplementary Figure 9 miR-378a-3p had no effect on LDLR-medicated LDL uptake in both HepG2 and Hepa1-6 cells. Two groups of HepG2 or Hepa1-6 cells were transfected with miR-378a-3p mimics or scramble (control). 48 hours after transfection, the intensity of fluorescence was quantified at 540/570 nm excitation/emission. Data represent mean ± SEM. NS: no significance (student *t* test)
